# Targeted delivery of black phosphorus nanosheets by ROS responsive complex hydrogel based on angiogenesis and antioxidant promotes myocardial infarction repair

**DOI:** 10.1186/s12951-024-02685-0

**Published:** 2024-07-22

**Authors:** Jiahui Zhang, Di Sun, Yishan Guo, Junran Tong, Qingyi Liu, Ran Gao, Yumiao Wei, Xiaopeng Guo

**Affiliations:** 1 Department of Cardiology, Union Hospital, Tongji Medical College, Huazhong University of Science and Technology, Wuhan, 430022 China; 2 Hubei Key Laboratory of Biological Targeted Therapy, Union Hospital, Tongji Medical College, Huazhong University of Science and Technology, Wuhan, 430022 China; 3 Hubei Engineering Research Center for Immunological Diagnosis and Therapy of Cardiovascular Diseases, Union Hospital, Tongji Medical College, Huazhong University of Science and Technology, Wuhan, 430022 China; 4 Department of Plastic Surgery, Union Hospital, Tongji Medical College, Huazhong University of Science and Technology, Wuhan, 430022 China; 5 Department of Cardiology, Binzhou Medical University Hospital, Binzhou, 256600 China; 6 Department of Radiology, Union Hospital, Tongji Medical College, Huazhong University of Science and Technology, Wuhan, 430022 China

**Keywords:** Myocardial infarction, Biomaterials, Black phosphorus nanosheets, ROS, Hydrogel

## Abstract

Myocardial infarction (MI) is one of the leading causes of death. This is attributed to the dramatic changes in the myocardial microenvironment post-MI. Therefore, effective intervention in the early stages of MI is significant for inhibiting its progression and improving cardiac function. Herein, an injectable composite hydrogel scaffold (Gel-pBP@Mg) was developed by integrating magnesium (Mg)-modified black phosphorus nanosheets (pBP@Mg) into a reactive oxygen species-responsive hydrogel (Gel). This loose and porous Gel provides a natural platform for carrying pBP@Mg. In situ, sustained release of pBP@Mg is achieved via responsive ROS degradation in the infarct site. The high ROS reactivity of Black phosphorus nanosheets (BPNSs) can effectively inhibit the progression of oxidative stress in the infarct area and reduce inflammatory response by down-regulating the NF-κB pathway. Additionally, the sustained release of Mg loaded on the surface of BPNSs can effectively promote angiogenesis in MI, which is significant for the long-term prognosis after infarction. Our developed Gel-pBP@Mg effectively blocked infarction progression and improved myocardial function by sustainably inhibiting the “oxidative stress-inflammation” reaction chain and pro-angiogenesis. This study reveals Gel-pBP@Mg composite therapeutic potential in treating MI through In vitro and In vivo studies, providing a promising modality for MI treatment.

**Graphical abstract:**

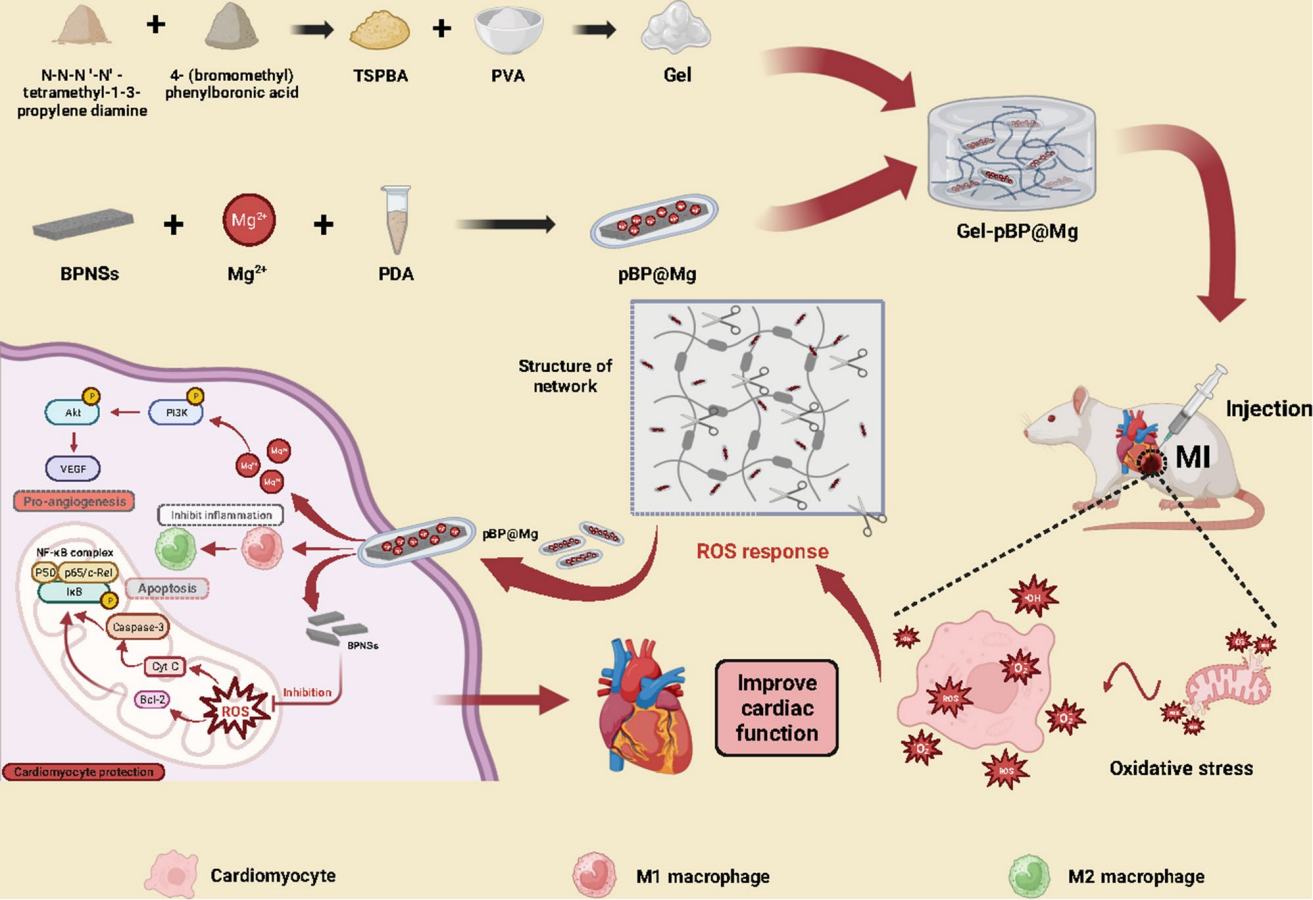

**Supplementary Information:**

The online version contains supplementary material available at 10.1186/s12951-024-02685-0.

## Introduction

Myocardial infarction (MI) threatens human life and health, typically resulting from blood flow blockage to the myocardium for various reasons. Cardiac cells lose blood perfusion post-MI, leading to an anoxic environment. When a dysfunction occurs in multiple mitochondria in cardiomyocytes, it can release massive reactive oxygen species (ROS) into the cytoplasm. This triggers cytochrome c (cyto-c) and activates the caspase signaling pathway, which can injure myocardial cells (CMs) [1, 2]. ROS can activate Toll-like receptor 4, which triggers NF-κB and induces inflammatory responses by producing chemokines and pro-inflammatory factors [3, 4]. Although existing clinical therapies can achieve myocardial reperfusion and reduce mortality, most studies have found myocardial necrosis and decreased cardiac function after treatment [5–7]. Percutaneous coronary intervention (PCI) is prone to vascular perforation, malignant arrhythmias and acute kidney injury. Lack of targeting of thrombolytic drugs can lead to cerebral hemorrhage [8]. More importantly, they all focus only on restoring blood flow to the infarct site and do not make effective treatments for oxidative stress and inflammatory responses in the infarct microenvironment. Therefore, there is an urgent need for an approach that can remodel the infarct microenvironment and remove the harm caused by MI from the root.

Black phosphorus nanosheets (BPNSs) are nanomaterials with a two-dimensional layered structure consisting of porous phosphorus atoms connected by strong P-P bonds within the layers and weak van der Waals forces between the layers [9]. It has become a significant biological material due to good biological compatibility, electrical conductivity, and thermal characteristics. BPNSs have a two-dimensional fold structure that provides a larger surface volume ratio and greater load capacity for superior drug delivery. Additionally, BPNSs possess strong ROS reactivity, making them an excellent choice to inhibit excessive oxidative stress [10]. Therefore, more studies have applied BPNSs in post-ischemic injury treatment, specifically in bone and neurovascular regeneration [11, 12]. However, the lone electron pair of phosphorus atoms makes BPNSs react with oxygen and water, limiting their biomedical application [13]. Dopamine (DA) is a biocompatible substance that can be oxidized and self-polymerized in an alkaline environment to form a super cohesive polydopamine (PDA) layer on the material surface. Accordingly, this property of PDA can be used to modify BP; the contact between oxygen or water and BPNSs can be isolated to a certain extent, thereby improving stability [14, 15]. Continuous ischemia and hypoxia post-MI are the main causes of myocardial injury; therefore, reducing oxidative stress-inflammatory storms may be insufficient to rescue the myocardium. Improving blood supply is crucial for promoting capillary formation and enhancing oxygen and nutrient supply to the infarct area. Clearance of metabolic waste can also inhibit MI progression [16, 17].

Furthermore, magnesium (Mg) can promote angiogenesis by enhancing vascular endothelial cell proliferation, adhesion, and motility and activating PI3K-Akt and MAPK pathways to promote VEGF and FGF release [18–20]. Mg can be anti-inflammatory by promoting M1 phenotypic macrophage transformation into M2 phenotypic macrophages [21]. However, only a few studies have investigated the efficacy of using Mg to treat MI. Consequently, we have used PDA modification and Mg surface loading to maximize the stability and biotherapy properties of BPNSs, achieve in situ sustained release post-MI, and improve microenvironment deterioration to inhibit cardiac obstruction progression.

Recently, with the in-depth understanding of the pathological changes of myocardial infarction microenvironment, using hydrogel biomaterials for local MI treatment has become a research hotspot. The therapeutic efficiency of drugs can be improved by the recently developed patches, hydrogels, and scaffolds [22–24]. Delivering nanomaterial to the MI site needs a safe, degradable, and high-loading rate drug carrier. Injectable hydrogels can be administrated locally to deliver drugs to the treatment site, avoiding the low targeting rate of intravenous administration. Additionally, the hydrogel can form a three-dimensional network structure with good molecular permeability and a high drug-loading rate [25–27]. Therefore, hydrogels are ideal materials for treating MI. Polyvinyl alcohol (PVA) is one of the most widely studied hydrogel materials; it is non-toxic, highly biocompatible, and has good mechanical properties. The hydroxy-rich chemical characteristics of PVA allow it to cross-link with various materials [28, 29]. Bio-responsive hydrogels have better controlled release ability and degradability than ordinary hydrogels. ROS-responsive hydrogels can be gradually decomposed in a high concentration of ROS environment, and a large amount of ROS generated in the infarcted area after infarction can decompose the ROS-responsive hydrogels to realize controlled release of drugs for the purpose of slow release of nanomaterials. [30–32].

**Fig. 1:**
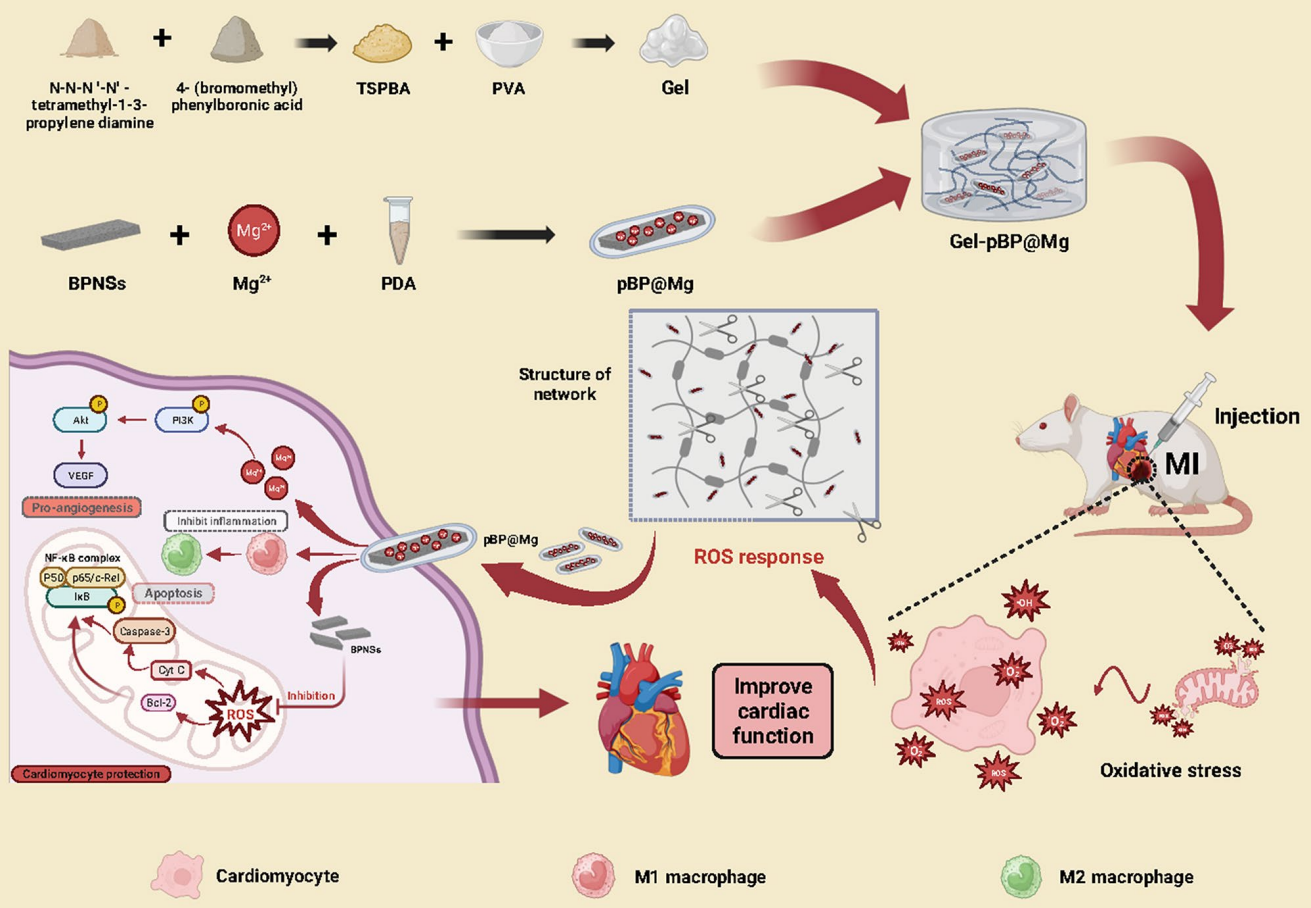
Schematic diagram of the synthesis and mechanism of Gel-pBP@Mg

Here, we designed a PVA-based ROS-responsive hydrogel (Gel) to achieve durable improvement of the infarct microenvironment after in situ injection of the composite hydrogel to inhibit post-MI progression by carrying surface Mg-loaded BPNSs **(**Fig. 1**).** Our in vivo and in vitro experiments showed that ROS released in the infarct area shortly post-MI could effectively decompose the Gel and promote the effective release of nanocomposites pBP@Mg in the Gel. The continuous ROS scavenging inhibits the oxidative stress-inflammatory reaction chain, promotes angiogenesis, improves blood supply in the infarction area, and effectively improves the microenvironment deterioration post-MI. It can significantly reduce myocardial tissue damage, improve myocardial function, and prevent the occurrence of adverse rain post-MI. These findings suggest that constructing composite hydrogels with multiple functions can significantly improve cardiac function post-MI.

## Results

### Synthesis and characterization of BPNSs and pBP@Mg

**Fig. 2:**
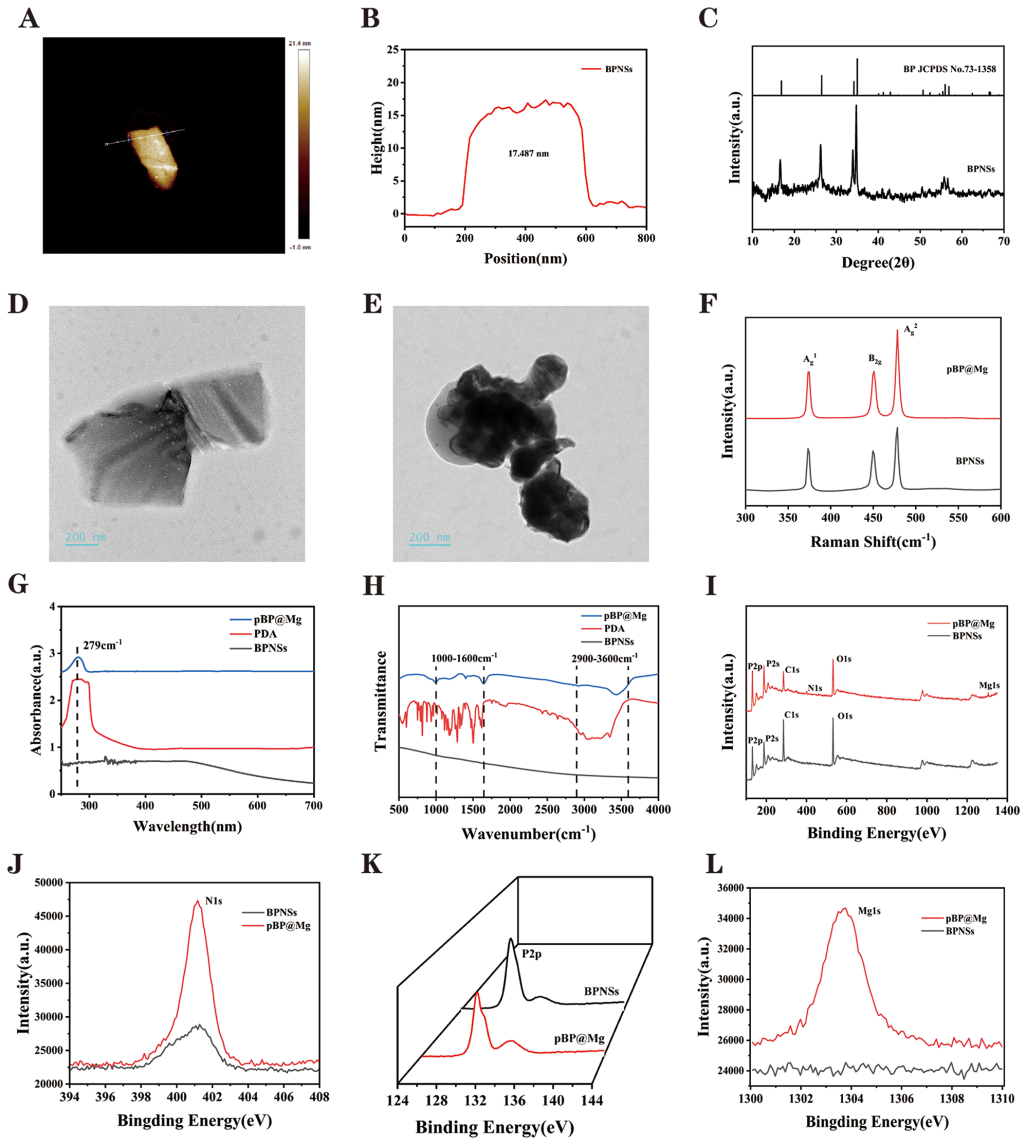
Characterization of physicochemical properties of BPNSs and pBP@Mg. (**A**) Representative AFM of BPNSs and (**B**) Thickness measurements. (**C**) Detection of XRD diffraction peaks of BPNSs compared with the standard black phosphorus block. (**D**) Representative TEM of BPNSs. (**E**) Representative TEM of pBP@Mg. (**F**) Absorption peaks of Raman spectra of BPNSs and pBP@Mg. (**G**) UV-vis detection of BPNSs, PDA, and pBP@Mg. (**H**) FTIR detection of BPNSs, PDA, and pBP@Mg. (**I**) Characteristic XPS absorption peaks of BPNSs and pBP@Mg. (**J**) XPS characteristic N1s absorption peaks of BPNSs and pBP@Mg. (**K**) XPS characteristic P2p absorption peaks of BPNSs and pBP@Mg. (**L**) XPS characteristic Mg1s absorption peaks of BPNSs and pBP@Mg

BPNSs were synthesized by removing bulk BP crystals using electrochemical stripping in a nonaqueous electrolyte. The resultant BPNSs were dispersed under ultrasound for 20 min. The BPNSs thickness was about 17 nm detected by Atomic Force Microscopy (AFM) and had a two-dimensional sheet structure **(**Fig. 2A and B**)**. X-ray diffraction (XRD) results showed that the absorption peak of BPNSs was high and sharp, and the crystallinity was good. The absorption peak of BPNSs coincided with that of the BP block of standard, indicating no new crystal lattice was formed **(**Fig. 2C**)**; then, the MgCl solution was mixed with BPNSs. Mg^2+^ forms coordination bonds (π bonds) with the lone pair of electrons of BPNSs and was loaded on BPNSs through strong chemical bonds and electrostatic adsorption. Modified by metal ions, the unpaired electrons of BPNSs form strong bonds, which increase the BPNSs’ stability [33–35]. Subsequently, Mg-loaded BPNSs and DA were added to the NaOH solution at pH = 8.5 to form PDA-wrapped pBP@Mg. Both bare BPNSs and PDA have negative charges, while Mg has positive charges. The Zeta potential confirmed that the surface potential of BPNSs undergoes the anticipated modifications, providing evidence of electrostatic adsorption between these components (Figure S1). TEM tests showed that the maximum lateral dimension of BPNSs and pBP@Mg was about 1–3 μm, presenting a standard multi-layer sheet structure. pBP@Mg was covered with a polymer layer of PDA (Fig. 2D–E), and Mg, oxygen (O), carbon (C), and phosphorus (P) were uniformly distributed on its surface (Figure S2).

Raman and Fourier transform infrared (FTIR) spectra were further tested. Raman spectroscopy results showed that both BPNSs and pBP@Mg presented three absorption peaks, A1g, B2g, and A2g, indicating that the modified BPNSs did not cause structural changes **(**Fig. 2F**)**. FTIR results showed that after PDA modification, pBP@Mg appeared in a series of absorption bands between 1200 and 1600 cm^–1^, which could be attributed to the aromatic ring and C-N bond in PDA. Additionally, the C-H and N-H stretching vibrations in PDA lead to the appearance of two new absorption bands between 2900 and 3600 cm^–1^. Therefore, the FTIR results proved the successful coating of BPNSs by PDA **(**Fig. 2H**)**. Then, the chemical composition of nanosheets was detected by X-ray photoelectron spectroscopy (XPS) **(**Fig. 2I and L**)**. The results showed that pBP@Mg detects the N1s peak at 400.6 eV, confirming the presence of a PDA layer. Weak N1s were detected in the bare BPNSs, possibly due to oxidation. The P2p peak intensity of bare BP (129.6 eV) was higher than that of pBP@Mg due to the absence of P element in the chemical structure of PDA. The Mg1 peak appeared in the 1305 eV, which successfully verified that the Mg^2+^ mixed with BPNSs. Therefore, these changes indicate that the modification of corresponding compounds was successful.

To verify that Mg and PDA modification enhanced the stability of BPNSs, UV-vis absorbance of BPNSs, PDA, and pBP@Mg was performed, showing strong light absorption of pBP@Mg at 279 nm due to PDA (Fig. 2G). The UV-vis absorbance of aqueous dispersions of BPNSs and pBP@Mg stored in the environment for 28 days was also examined. After 28 days, BPNSs absorbance decreased more than pBP@Mg (Figures S3–S4). The results provided strong evidence that adding Mg and PDA improves the stability of BPNSs.

### Synthesis and characterization of composite hydrogel

**Fig. 3:**
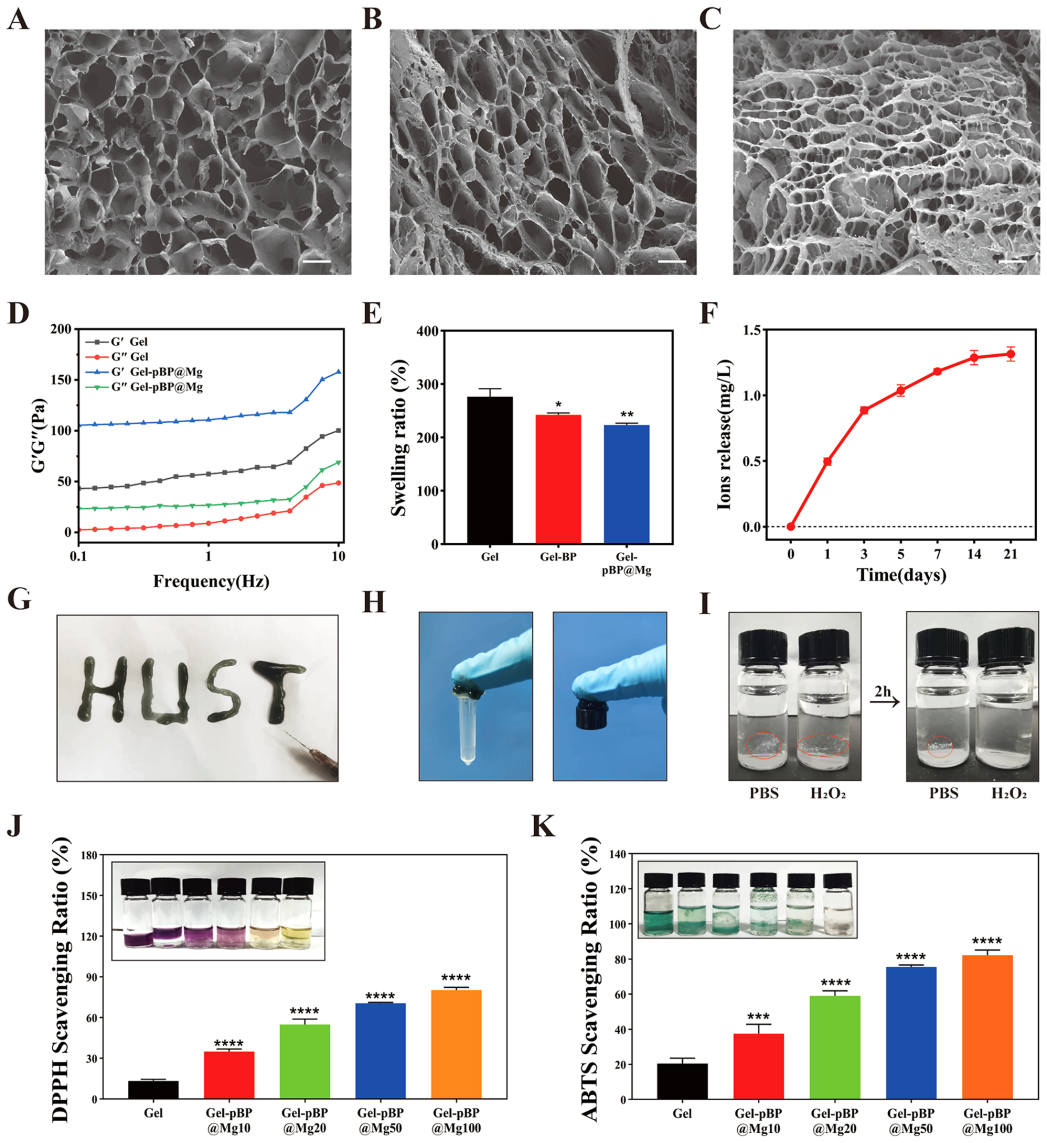
The physical and chemical properties of Gel characterization. Representative SEM of Gel (**A**), Gel-BP (**B**), and Gel-pBP@Mg (**C**). (**D**) Measurement of Gel and Gel-pBP@Mg rheological properties. (**E**) Gel, Gel-BP, and Gel-pBP@Mg swelling performance testing (*n* = 3). (**F**) Gel-pBP@Mg slow-release capacity assay. (**G**)Injectability of Gel-pBP@Mg. (**H**) The adhesiveness of Gel-pBP@Mg. (**I**) In vitro ROS responsiveness of the Gel. (**J**) DPPH reactive oxygen species scavenging ability of Gel with different concentrations of Gel-pBP@Mg (*n* = 3). (**K**) ABTS reactive oxygen species scavenging ability of Gel with different concentrations of Gel-pBP@Mg (*n* = 3). Scale: 10 μm. *p < 0.05; **p < 0.01; ***p < 0.001; ****p < 0.0001

ROS-responsive hydrogels were obtained by a two-step method. The hydrogel precursor was synthesized by the quaternization of N-N-N’-N’-tetramethyl-1-3-propylene diamine with excess 4- (bromomethyl) phenylboronic acid and then mixed with PVA. The boric acid bond of the hydrogel precursor could quickly cross-link with the hydroxyl group on PVA to form a borate ester bond, forming Gel. Gel-pBP@Mg and Gel-BP composite hydrogels were prepared by adding pBP@Mg and BPNSs to the hydrogel precursor and mixing them with PVA. SEM examination of the cross-sections of hydrogels after lyophilization showed that all hydrogels had loose porous structures **(**Fig. 3A and C**)**. Compared with Gel, Gel-pBP@Mg showed more cross-linked structures and higher porosity. This may be due to the rapid formation of hydrogen bonds between pBP@Mg and PVA by adding PDA, forming a more interconnected network [36].

Furthermore, the rheological properties of hydrogel were examined by frequency scanning method with fixed amplitude at 0.1–10 Hz (Fig. 3D). All composite hydrogels maintained nonlinear rheological behavior. The storage modulus (G’) was higher than the loss modulus (G”), indicating that all tested hydrogels exhibited similar behavior to elastic solids. Upon adding pBP@Mg, both the G’ and G” increased, which might be due to the hydrogen bond formed between them. Swelling is another property of hydrogels that directly affects their drug-loading properties (Fig. 3E and S5). The test results showed that the swelling performance of Gel-BP and Gel-pBP@Mg decreased slightly compared with Gel due to the smaller pore size of the Gel caused by pBP@Mg addition. To ensure that Gel-loaded pBP@Mg could be released slowly at the infarct site, we examined the slow-release ability of Gel-pBP@Mg (Fig. 3F). On days 1, 3, 5, 7, 14, and 21 of immersion in PBS, the content of p-elements gradually increased and remained slowly rising on day 21. This indicates that Gel-pBP@Mg is capable of achieving long-term slow release of pBP@Mg. The Gel also revealed physical properties suitable for research. The mode of administration of an intramyocardial injection depended on its injectability and adhesion (Fig. 3G and H, Movie S1). The composite hydrogel injected locally had a suitable electrical conductivity for the myocardium and avoided functional damage caused by local use (Figure S6).

### The antioxidant capacity of Gel-pBP@Mg

To investigate the ROS-responsive degradation behavior of Gel-pBP@Mg, the same volume of Gel-pBP@Mg was placed into sample flasks containing H_2_O_2_ and PBS for observation **(**Fig. 3I**)**. After 24 h, Gel-pBP@Mg in the sample bottle of H_2_O_2_ was completely degraded, and the volume of Gel-pBP@Mg in PBS was slightly reduced. This is because in the high-concentration ROS environment, the borate ester bond in Gel is oxidized and broken, and the hydrogel gradually decomposed to achieve the drug-controlled release. This suggests that Gel-pBP@Mg has good ROS responsiveness.

Subsequently, we synthesized different concentrations of Gel-pBP@Mg and used Gel-pBP@Mg10, Gel-pBP@Mg20, Gel-pBP@Mg50, and Gel-pBP@Mg100 to represent the Gel containing 10, 20, 50, and 100 µg mL^–1^ of pBP@Mg, respectively. To further investigate the antioxidant properties of Gel-pBP@Mg composites, the antioxidant properties of composite hydrogels with different concentrations were evaluated using 2, 2-diphenyl-1 -(2,4, 6-trinitrobenzene) hydrazine (DPPH) detection kit **(**Fig. 3J**)**. DPPH is a stable reactive nitrogen species (RNS) compound whose ethanol solution is dark purple and decolorizes in the presence of antioxidants. The same volume of composite hydrogel was put into the sample bottle, and the same volume of DPPH/ ethanol solution was added. The results showed that the composite hydrogels could decolorize DPPH, and the effect was more significant with the increased concentration. Ultraviolet absorbance analysis of 517 nm supernatant showed that the clearance rate of Gel-pBP@Mg100 reached 80.3%.

Further, we used ABTS free radical scavenging ability detection kit to test the antioxidant activity of the composite **(**Fig. 3K**)**. ABTS was oxidized to form a stable blue-green cationic ABTS radical with a maximum absorption peak at 405 nm. Upon adding the tested substance to the ABTS free radical solution, the antioxidant components reacted with the ABTS free radical and made the reaction system fade. The results showed that the fading of the reaction system was significant, and the ABTS clearance rate of Gel-pBP@Mg100 reached 82.2%. The above experiments verify that the Gel-pBP@Mg composite has ROS responsiveness and excellent antioxidant ability.

### In vitro Gel-pBP@Mg removal ability of ROS

**Fig. 4:**
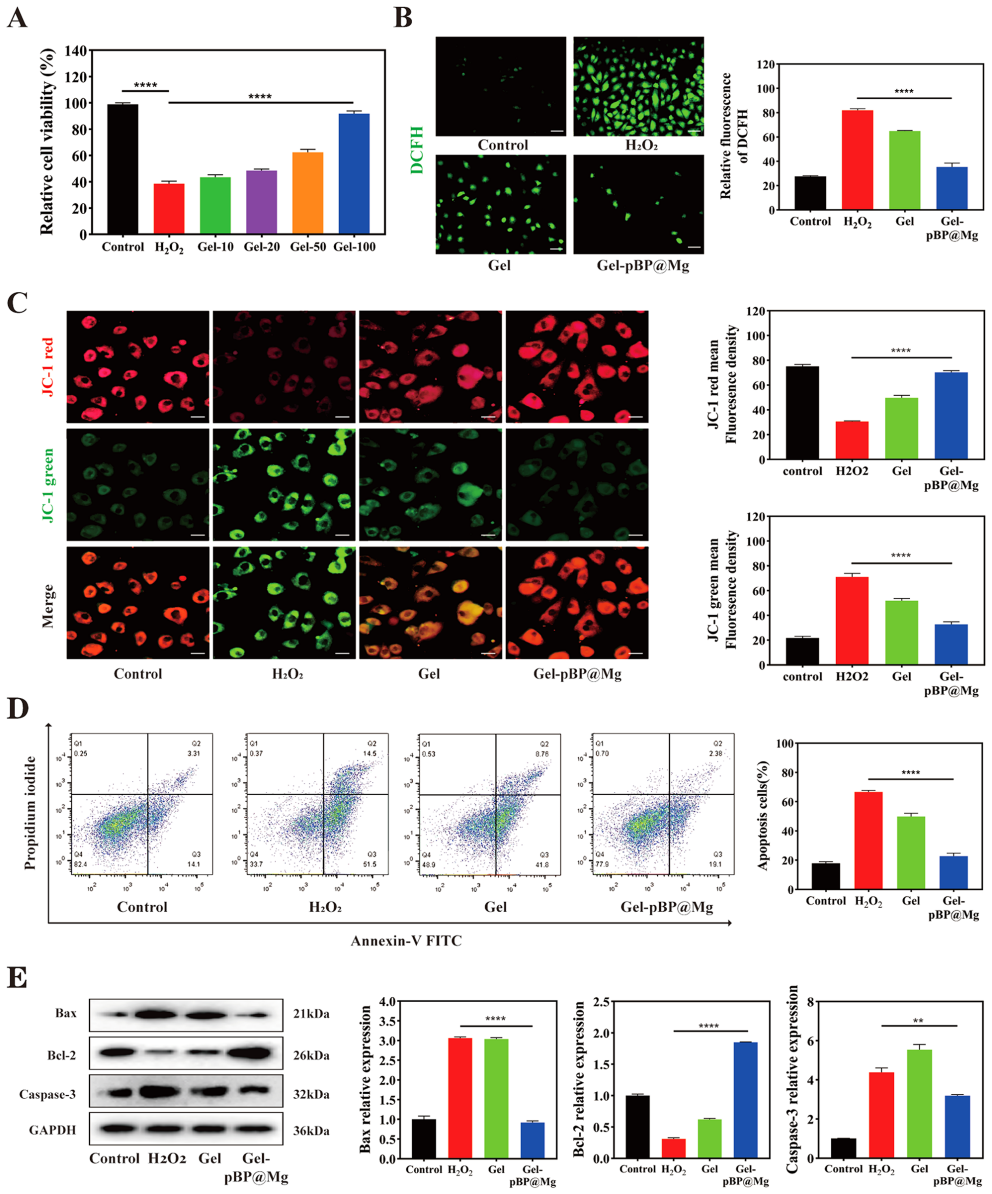
In vitro safety, antioxidant capacity and apoptosis inhibition of composite hydrogels. (**A**) Relative viability of CMs treated with different concentrations of Gel-pBP@Mg. All treatments groups were performed in the presence of H_2_O_2_ (*n* = 3). (**B**) DCFH-DA fluorescent staining and quantitative statistical analysis of Control, H_2_O_2_, Gel + H_2_O_2_, and Gel-pBP@Mg + H_2_O_2_ (*n* = 4). Scale: 100 μm. (**C**) JC-1 fluorescence staining of control, H_2_O_2_, Gel + H_2_O_2,_ and Gel-pBP@Mg + H_2_O_2_. All treatments groups were performed in the presence of H_2_O_2_ (*n* = 4). Scale: 25 μm. (**D**) Apoptosis was analyzed by flow cytometry with Control, H_2_O_2_, Gel + H_2_O_2_, and Gel-pBP@Mg + H_2_O_2_ and quantitative statistical analysis. All treatments groups were performed in the presence of H_2_O_2_ (*n* = 3). (**E**) Detection and quantitative statistical analysis of apoptosis-related protein expression by WB. All treatments groups were performed in the presence of H_2_O_2_ (*n* = 3). *p < 0.05; **p < 0.01; ***p < 0.001; ****p < 0.0001

The cell viability of rat H9C2 CMs treated with Gel-pBP@Mg at different concentrations in the presence of H_2_O_2_ was measured by Cell Counting Kit-8 (CCK-8) **(**Fig. 4A**)**. Different concentrations of Gel-pBP@Mg showed excellent anti-oxidative stress effects caused by H_2_O_2_. The maximum concentration of 100 µg mL^− 1^ Gel-pBP@Mg had the most significant effect, close to that of the control group. Therefore, the concentration of 100 µg mL^− 1^ was subsequently selected to test the ability of the composite hydrogel to remove ROS.

To evaluate the biocompatibility necessary for in vivo application, we tested the viability of cells cultured on the complex hydrogel by CMs. CCK-8 test showed an insignificant difference between Gel and Gel-pBP@Mg and control groups (Figure S7). Calcein-AM could cross the cell membrane into living cells with low toxicity. The cell density increased after 48 h culture compared with 24 h. The control, Gel, and Gel-pBP@Mg groups showed similar cell density and morphology differences. This indicates that all hydrogels have good biocompatibility (Figure S8). This is because the porous structure of hydrogels and the fold structure of BPNSs provide cell adhesion sites, and the composite hydrogels are prepared with non-toxic materials.

Dichloro-dihydro-fluorescein diacetate (DCFH-DA) was able to cross the cell membrane and react with intracellular ROS to produce products with green fluorescence. Therefore, we evaluated the level of intracellular superoxide anion and hydroxyl radical scavenging by composite hydrogel treatment using DCFH-DA staining. (Figs. 4B). The fluorescence intensity of CMs incubated with Gel and Gel-pBP@Mg was significantly lower than that of the H_2_O_2_ treatment group, indicating that all hydrogel groups could remove ROS from CMs. The fluorescence intensity of the Gel-pBP@Mg group was similar to that of the control group, which fully proved that the complex hydrogel could effectively inhibit the oxidative stress of CMs.

### Role of Gel-pBP@Mg in ROS injury to cardiomyocytes in vitro

At high ROS levels, the structure and catalytic capacity of cyto-c undergo significant changes, transforming into a peroxidase that catalyzes cardiolipin (CL) peroxidation, causing CL peroxidation and cyto-c dissociation. The release of cyto-c into the cytoplasm promotes the caspase signaling pathway activation, thus triggering the apoptotic cascade [37, 38]. The decline of mitochondrial membrane potential is a landmark event in the early stage of apoptosis. The mitochondrial membrane potential of CMs in different treatment groups was measured with a JC-1 detection kit **(**Fig. 4C**)**. The green fluorescence of the H_2_O_2_ group was the strongest, indicating that mitochondrial membrane potential decreased, and mitochondrial damage caused by ROS was the strongest. The green fluorescence intensity of Gel-pBP@Mg was similar to or even slightly lower than that of the control group. The JC-1 test results proved that Gel-pBP@Mg could alleviate the mitochondrial damage of CMs.

Apoptosis flow cytometry was performed to demonstrate ROS-induced CM apoptosis further **(**Fig. 4D**)**. The CMs in the upper right quadrant (late apoptosis) and lower right quadrant (early apoptosis) are apoptotic cells. Data showed that the CM cell apoptosis rate in the H_2_O_2_ group was 66.64% ± 0.90%, significantly increasing compared with the control group. Additionally, the H_2_O_2_-induced percentage of apoptotic CM cells in the Gel-pBP@Mg group was significantly decreased (22.80% ± 1.64%) compared with the H_2_O_2_ group. These data suggest that Gel-pBP@Mg can reduce H_2_O_2_-induced apoptosis of CM cells.

We used western blot (WB) to analyze the expression of apoptosis-related molecules to investigate the apoptosis mechanism of CMs further **(**Fig. 4E**)**. The expressions of pro-apoptotic molecules Bax and Caspase-3 were significantly increased in the H_2_O_2_ group while significantly decreasing in the Gel-pBP@Mg group. Bcl-2 expression in the Gel-pBP@Mg group was significantly increased after incubation compared with the H_2_O_2_ group. The results showed that Gel-pBP@Mg treatment increased mitochondrial activity and inhibited apoptosis of CMs.

### Gel-pBP@Mg exerts anti-inflammatory effects by promoting polarization of RAW264.7 towards the M2 phenotypic phenotype

MI generates a large amount of ROS, and the ensuing large amount of proinflammatory factors induces macrophage polarization to the M1 phenotype, which leads to an increased inflammatory response in the infarcted area. Therefore, it is particularly important to administer anti-inflammatory therapy at the early stage of myocardial infarction as an effective way to prevent further myocardial death.

**Fig. 5:**
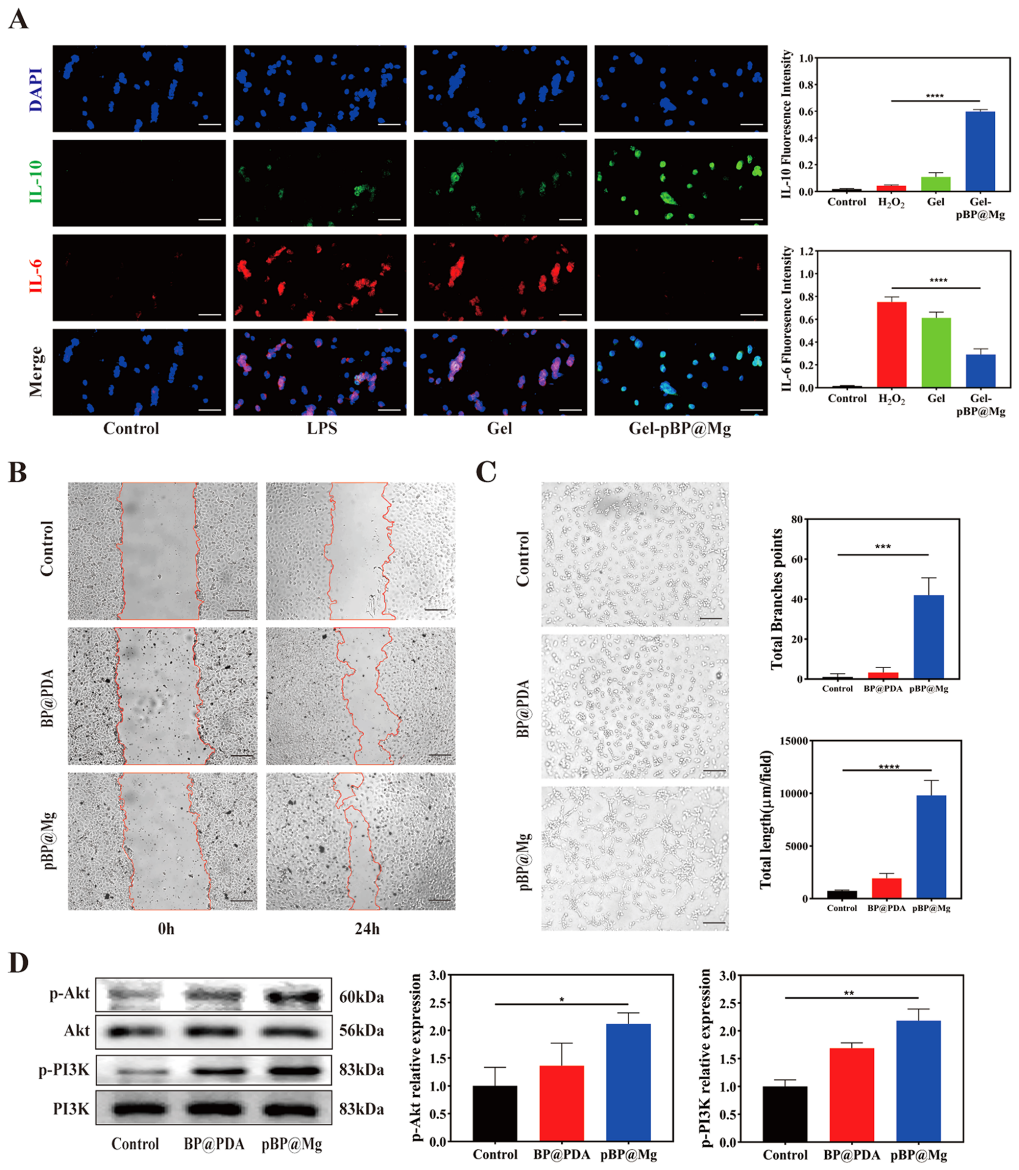
In vitro anti-inflammatory and pro-HUVEC migration and tube formation capacity of Gel-pBP@Mg. (**A**) Control, H_2_O_2_, Gel, and Gel-pBP@Mg promote polarization of RAW264.7 toward the M2 phenotype (*n* = 4). Scale: 10 μm. (**B**) control, BP@PDA, and pBP@Mg incubation for 24 h to promote HUVEC migration and quantitative statistical analysis (*n* = 3). Scale: 100 μm. (**C**) control, BP@PDA, and pBP@Mg incubation for 6 h to promote HUVEC tube formation and quantitative statistical analysis (*n* = 3). Scale: 10 μm. (**D**) Quantitative statistical analysis of the pro-angiogenesis expression associated protein by WB (*n* = 3). *p<0.05; **p<0.01; ***p<0.001; ****p<0.0001

We examined the anti-inflammatory activity of Gel-pBP@Mg complex hydrogels with RAW264.7. RAW264.7 induced macrophage polarization to the M1 phenotype by LPS, followed by treatment with either Gel or Gel-pBP@Mg. Immunofluorescence co-localization of IL-6 and IL-10 showed that the expression of inflammatory IL-6 was significantly reduced in the Gel-pBP@Mg-treated group. indicating that Gel-pBP@Mg effectively promoted the polarization of M1 phenotype macrophages to M2 phenotype (Fig. 5A). In addition, the supernatants of RAW264.7 cell cultures from different treatment groups were collected, and the expressions of TNF-α and TGF-β were detected by ELISA (Figure S9). The expression level of TNF-α in M1 phenotypic macrophages in the LPS group was significantly elevated, whereas the expression level of TGF-β was the highest in M2 phenotypic macrophages in the Gel-pBP@Mg group. The results indicated that Gel-pBP@Mg treatment significantly decreased the polarization of M1 phenotypic macrophages, which in turn promoted the polarization of M2 phenotypic macrophages toward anti-inflammatory macrophages.

### Gel-pBP@Mg promotes HUVEC migration and tube formation

The migration and proliferation of endothelial cells play an important role in injury repair. Post-MI, the blood vessels that begin to grow in the marginal area gradually extend to the infarct core. A network of capillaries delivers oxygen to the infarct area, facilitating nutrient exchange and waste removal and preventing further CM death. To verify that the composite promotes endothelial cell migration and tubular formation, we tested the function of BP@PDA and pBP@Mg in HUVEC. After 24 h incubation, HUVEC mobility in pBP@Mg and BP@PDA groups reached 70.94% and 51.18%, respectively, and the center of scratches in pBP@Mg group had realized admixture (Fig. 5B and S10). The tube-forming experiment further confirmed these results. After 6 h incubation, the number of branches of the pBP@Mg tube reached 62, and the forming length of the tube reached 9803.33 μm (Fig. 5C).

Mg^2+^ can be taken up by cells through the ion channel function protein TRPM7 and then exert its enzymatic reaction. Deleting the TRPM7 kinase domain can defect Mg^2+^ absorption in the mouse intestine [39]. Mg^2+^ can activate PI3K phosphorylation, activating Akt and increasing VEGF expression to promote angiogenesis [40]. Therefore, we detected p-PI3K and p-Akt expression in treated HUVEC cells through WB (Fig. 5D). The p-PI3K and p-Akt expression were significantly increased in the pBP@Mg group. The BP@PDA group also had a vasotropic effect, possibly due to the excellent electrical conductivity of BPNSs that enhances cell-cell connections [41].

### Gel-pBP@Mg inhibits myocardial apoptosis through antioxidant stress

**Fig. 6:**
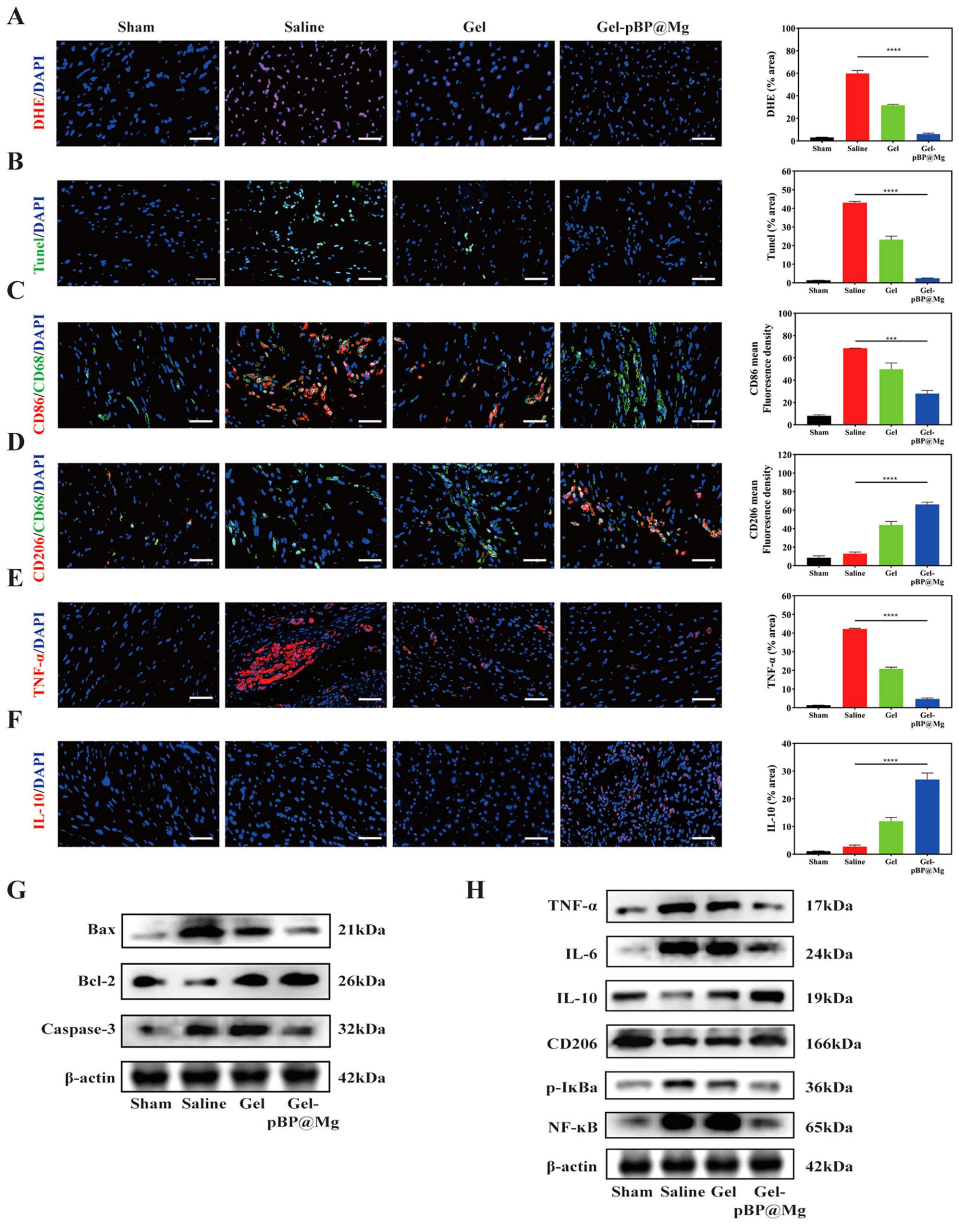
Gel-pBP@Mg exerts anti-oxidative stress, attenuates apoptosis and promotes M2 phenotypic macrophage polarization in MI rats. (**A**) DHE immunofluorescence staining and quantitative statistical analysis on day 3 of modeling in sham, saline, Gel, and Gel-pBP@Mg groups (*n* = 4). (**B**) Tunel immunofluorescence staining and quantitative statistical analysis on day 3 of modeling in sham, saline, Gel, and Gel-pBP@Mg groups (*n* = 4). (**C**) CD86/CD68 immunofluorescence co-localization staining and quantitative statistical analysis on day 3 of modeling in sham, saline, Gel, and Gel-pBP@Mg groups (*n* = 4). (**D**) CD206/CD68 immunofluorescence co-localization staining and quantitative statistical analysis on day 3 of modeling in sham, saline, Gel, and Gel-pBP@Mg groups (*n* = 4). (**E**) TNF-α immunofluorescence staining and quantitative statistical analysis on day 3 of modeling in sham, saline, Gel, and Gel-pBP@Mg groups (*n* = 4). (**F**) IL-10 immunofluorescence staining and quantitative statistical analysis on day 3 of modeling in sham, saline, Gel, and Gel-pBP@Mg groups (*n* = 4). (**G**) WB detection of apoptosis pathway-related protein expression (*n* = 3). (**H**) WB detection of inflammatory factor proteins and NF-κB inflammatory pathway-related protein expression (*n* = 3). Scale: 10 μm. *p<0.05; **p<0.01; ***p<0.001; ****p<0.0001

Post-MI, the damaged mitochondria release ROS, inducing cardiomyocyte apoptosis. To observe the effect of Gel-pBP@Mg on ROS levels in the infarcted area, we constructed a rat myocardial infarction model and injected hydrogels in situ, and rat hearts were collected on day 3 after myocardial infarction for DHE immunofluorescence staining (Fig. 6A). Quantitative analysis of the ROS signal found that the ROS signal in the Gel-pBP@Mg group decreased from 59.96 to 6.13% compared with the normal saline group. These data support that Gel-pBP@Mg can significantly inhibit ROS generation in infarct tissue.

Several ROS can activate the Caspase signaling pathway and induce apoptosis. Therefore, we examined Caspase-3 and Tunel to assess apoptosis levels in the infarct area (Fig. 6B and S11). Caspase-3 and Tunel results confirmed that antioxidant stress mitigated the damage caused by ROS and prevented further damage to cardiomyocytes. The Caspase-3 and Tunel signals in the Gel-pBP@Mg group were 5.37% and 2.54%, respectively. The Gel group showed decreased Caspase-3 and Tunel signals, potentially due to its ROS sensitivity.

To further verify that the composite material alleviated the apoptosis of cardiomyocytes, we evaluated protein expression in the classical apoptosis pathway through the WB experiment (Fig. 6G and S12). The results showed that the protein expression of the Gel-pBP@Mg group was similar to that of the sham group. Compared with the saline group, the pro-apoptotic Bax and Caspase-3 protein expressions were significantly decreased, while the anti-apoptotic Bcl-2 protein expression was increased. These findings suggest that injecting Gel-pBP@Mg into the infarction area can reduce ROS levels and alleviate myocardial apoptosis through its antioxidant stress effect. Gel-pBP@Mg was validated to enhance myocardial activity and provide therapeutic benefits.

### Gel-pBP@Mg inhibits the inflammatory response in the infarction area

Studies have shown that 1–3 days after the onset of myocardial infarction, circulating monocytes infiltrate into the tissue and differentiate into inflammatory macrophages. Subsequently, the transition from the inflammatory to the reparative phase occurs 3–5 days after infarction. As pro-inflammatory cells, M1 phenotypic macrophages lead to a massive inflammatory infiltration in the infarcted area. In contrast, M2 phenotypic macrophages are inflammation-suppressive cells, and increased numbers help to alleviate the abnormal inflammatory response in the infarcted area. We assessed the level of macrophage expression in the infarcted area by immunofluorescence co-localization staining for CD86/CD68 and CD206/CD68 (Fig. 6C and D). M1 phenotypic macrophage aggregation was significantly reduced in the Gel-pBP@Mg group compared to the large number of M1 phenotypic macrophages in the saline group. On the contrary, M2 phenotypic macrophages were heavily aggregated in the Gel-pBP@Mg group. This suggested that Gel-pBP@Mg had an inhibitory effect on the progression of post-infarction inflammation. Next, we detected the expression levels of tumor necrosis factor-alpha (TNF-α), and IL-10 (Fig. 6E and F). TNF-α expression by M1 phenotypic macrophages was higher in the saline group, while IL-10 expression by M2 phenotypic macrophages was higher in the Gel-pBP@Mg group. These show that the complex hydrogel treatment promoted M1 phenotypic macrophage transformation into M2 phenotypic macrophages and alleviated the abnormal inflammatory response in the infarction area.

WB was conducted to evaluate further the protein expression to validate this result (Fig. 6H and S13). As we expected, the expression of related proteins showed significant differences. The expression of pro-inflammatory factors TNF-α and IL-6 was suppressed in the Gel-pBP@Mg group, whereas the expression of the anti-inflammatory factors IL-10 and CD206 was significantly decreased in the saline group. In addition, p-IκBα and NF-κB were abundantly expressed in the saline group, whereas their expression was significantly reduced in the Gel-pBP@Mg group. These results support the ability of Gel-pBP@Mg to exert an inhibitory inflammatory response by promoting macrophage conversion to the M2 phenotype. The potential therapeutic role of composite hydrogel in inhibiting inflammation was demonstrated.

### Gel-pBP@Mg can improve cardiac function and alleviate fibrosis in MI rats

**Fig. 7:**
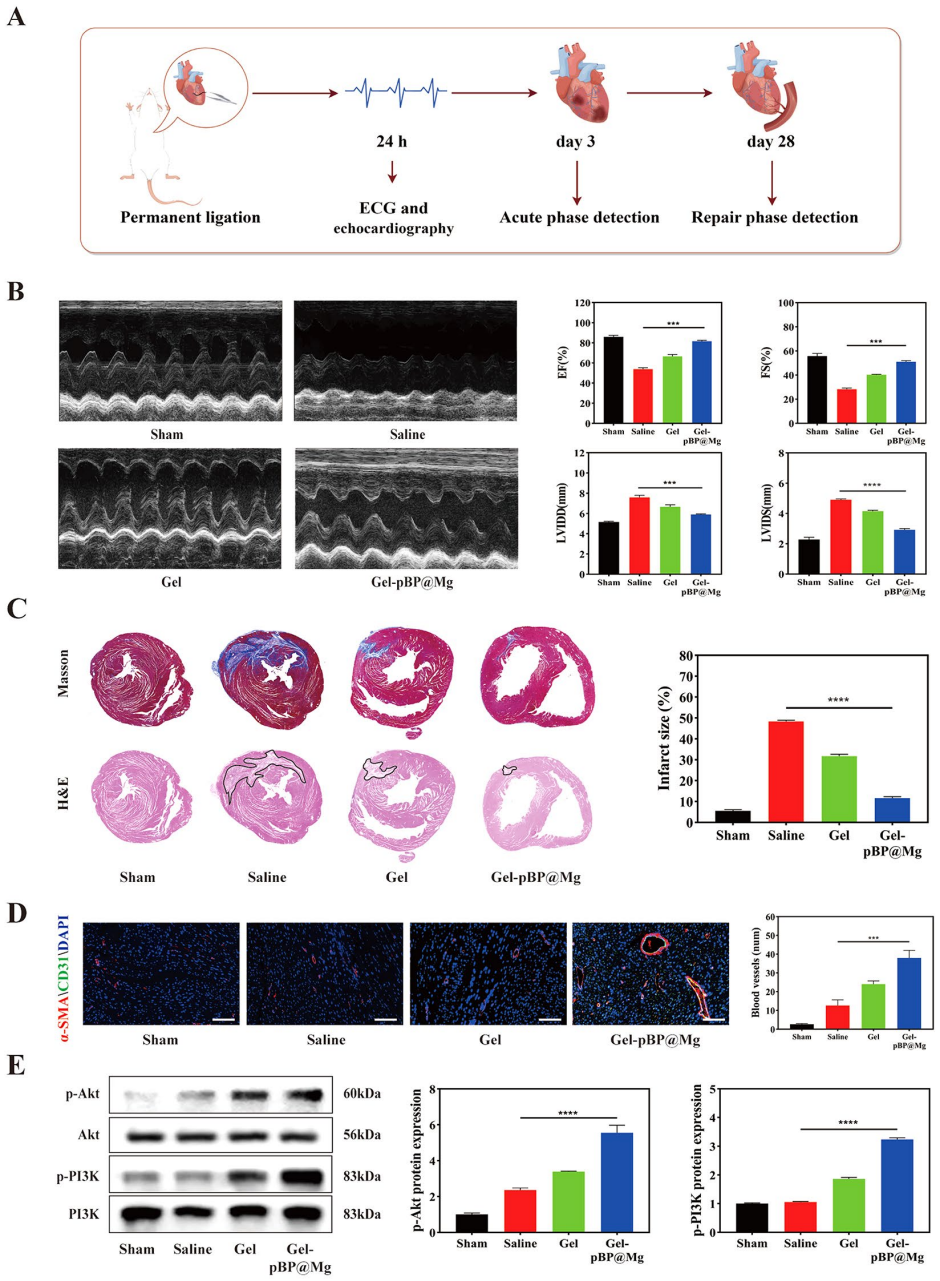
Gel-pBP@Mg improved cardiac function and myocardial fibrosis and promotes neoangiogenesis in MI rats. (**A**) Experimental timeline of SD rats treated with composite hydrogel. (**B**) Echocardiography was performed on day 28 and EF, FS, LVIDD, and LVIDS were quantitatively analyzed in sham, saline, Gel, and Gel-pBP@Mg groups (*n* = 5). (**C**) Masson and H&E staining on day 28 heart tissue sections in sham, saline, Gel, and Gel-pBP@Mg groups and quantitative statistical analysis of MI area (*n* = 3). (**D**) α-SMA/CD31 immunofluorescence co-localization staining and quantitative statistical analysis of new blood vessels on day 28 of modeling in sham, saline, Gel, and Gel-pBP@Mg groups (*n* = 5). (**E**) WB detection and quantitative statistical analysis of PI3K-Akt promote angiogenic pathways related to protein expression (*n* = 3). Scale: 10 μm. *p<0.05; **p<0.01; ***p<0.001; ****p<0.0001

The rat MI model was constructed by ligating the left anterior descending branch of the coronary artery with thoracotomy. After blocking coronary blood flow, the experimental group received an intramyocardial injection of Gel or Gel-pBP@Mg immediately (Fig. 7A). An electrocardiogram was performed within 24 h after surgery (Figure S14). The ECG showed obvious ST segment elevation, which confirmed the success of the MI model.

Immediately post-MI, damage-associated molecular patterns (DAMPs) are activated, which trigger inflammation by activating pro-inflammatory signaling pathways such as toll-like receptors and IL-1, leading to myocardial death, wall thinning, and ventricular dilation. Subsequently, fibroblasts promote collagen synthesis to form scar tissue. Meanwhile, angiogenesis in the infarct tissue was inhibited, and ventricular remodeling occurred. This process is completed approximately four weeks post-MI [4, 42, 43]. Therefore, echocardiography was performed on day 28 post-MI to detect cardiac function (Fig. 7B). Compared with the saline group, the cardiac function of the Gel-pBP@Mg group was significantly improved. After four weeks of intervention, ejection fraction (EF) of 81.63% and fractional shortening (FS) of 51.16% were improved with time in the MI + Gel-pBP@Mg group, and ventricular systolic function (LVIDD = 5.90 mm, LVIDS = 2.92 mm) was saved.

Fresh heart tissue was obtained from rats on day 28 after MI for tissue sectioning. Triphenyl tetrazolium chloride (TTC) staining showed that MI was serious in the saline group, and the MI area was reduced in the Gel-pBP@Mg group (Figure S15). The degree of myocardial fibrosis and collagen deposition was then assessed by Masson trichromatic and hematoxylin and eosin (H&E) staining (Fig. 7C). The results showed that after Gel and Gel-pBP@Mg treatment, the fibrosis and collagen deposition were alleviated, and the infarct size was significantly reduced. Compared with the saline group, the infarct size in the Gel-pBP@Mg group was reduced from 48.40 to 11.65%. These findings strongly support that Gel-pBP@Mg can improve ventricular remodeling after MI and reduce myocardial fibrosis.

Ischemia and hypoxia in the infarction area are unconducive to myocardial repair. therefore, promoting angiogenesis in the infarction area is the key to improving myocardial viability. The neovascularization brings blood, nutrients, and oxygen to the infarct site and removes metabolic waste. We evaluated neovascularization in MI area through alpha smooth muscle actin (𝛼-SMA) and CD31 (Fig. 7D). Immunofluorescence co-localization analysis showed that 𝛼-SMA and CD31 expressions in the Gel-pBP@Mg group were significantly increased, and their angiogenesis-promoting effects were significant. Compared with the saline group, the number of new vessels in the Gel-pBP@Mg group increased by more than three times, consistent with WB (Fig. 7E). PI3K and Akt proteins in the pro-angiogenesis pathway were phosphorylated, activated, and significantly expressed in the Gel-pBP@Mg group. These results suggest that Gel-pBP@Mg activates the PI3K-Akt pathway at the infarction site, promotes neovascularization, and helps alleviate myocardial injury.

### Gel-pBP@Mg inhibits apoptosis and promotes angiogenesis through the NF-κB and PI3K-Akt pathways

**Fig. 8:**
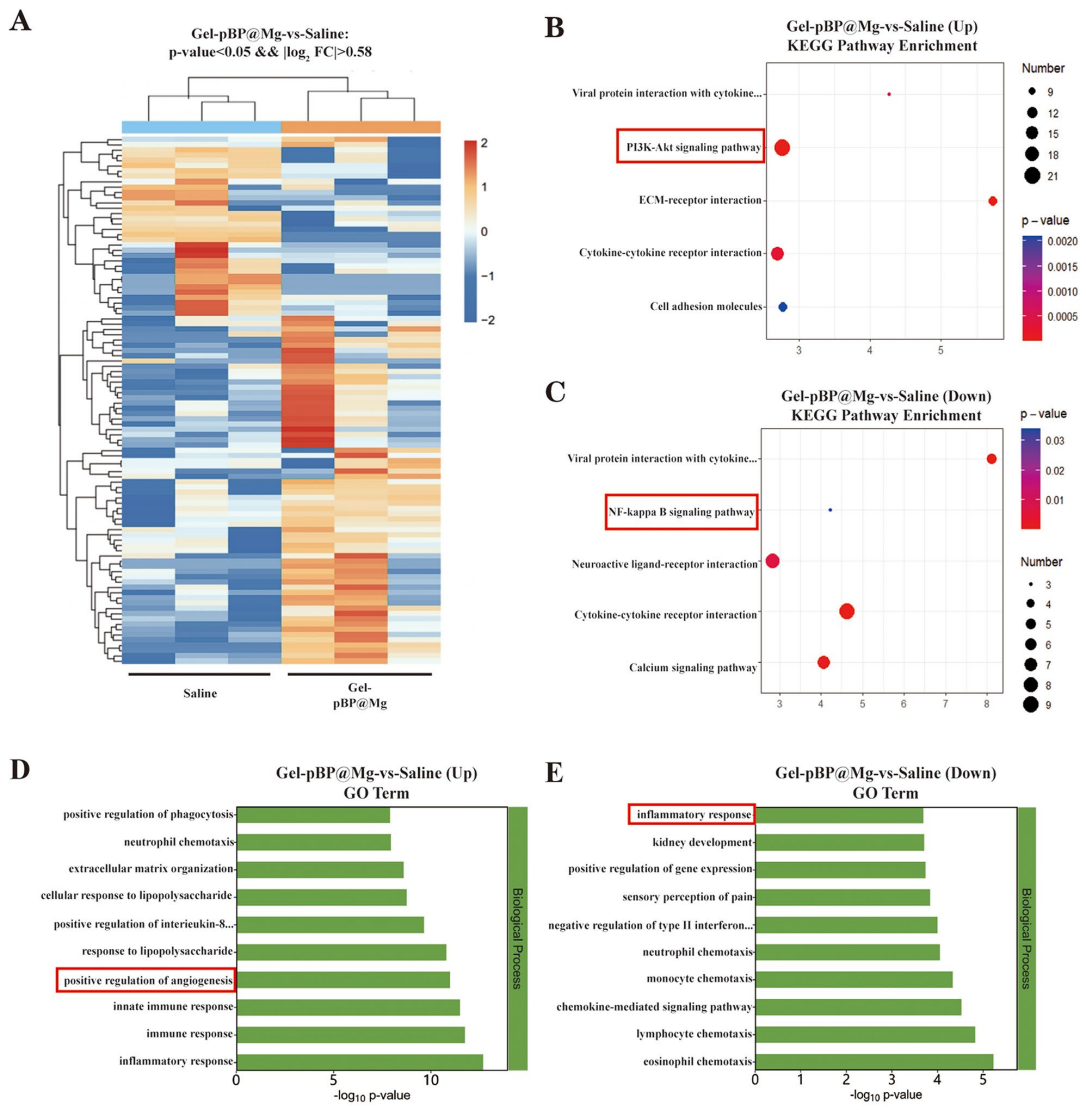
Transcriptome sequencing and KEGG and GO enrichment analysis. (**A**) Heat maps of gene expression in saline and Gel-pBP@Mg group. (**B**–**C**) KEGG enrichment analysis revealed up-regulated and down-regulated pathways in the Gel-pBP@Mg group compared to the saline group. (**D**–**E**) GO enrichment analysis showed the biological processes of up-regulated and down-regulated expression in the Gel-pBP@Mg group compared with the saline group

Gel-pBP@Mg could improve heart function and reduce infarct size in vivo. To further explore the therapeutic mechanism and biological process of Gel-pBP@Mg at the infarction site, fresh heart tissues from the saline group and Gel-pBP@Mg group were analyzed by transcriptome sequencing, and the results were analyzed for KEGG and GO enrichment. Heat maps of the saline group and Gel-pBP@Mg group showed differences in gene expression patterns between the two groups **(**Fig. 8A**)**. Then, the enrichment analysis of the KEGG pathway was performed **(**Fig. 8B and C**)**. Compared with the saline group, the PI3K-Akt signaling pathway expression was increased in the Gel-pBP@Mg group, while the NF-κB signaling pathway expression was decreased. GO enrichment analysis showed that these differential genes were enriched in pathways promoting angiogenesis, inflammatory response, and immune response **(**Fig. 8D and E**)**. The Gel-pBP@Mg group showed up-regulation of pro-angiogenesis genes, while inflammation and immune response genes were enriched in the saline group. These results suggest that Gel-pBP@Mg inhibits myocardial apoptosis and promotes angiogenesis in the infarction area through the NF-κB and PI3K-Akt pathways. Appropriate experiments were conducted to validate these results.

### Gel and Gel-pBP@Mg implantation did not cause damage to other organs of rats

**Fig. 9:**
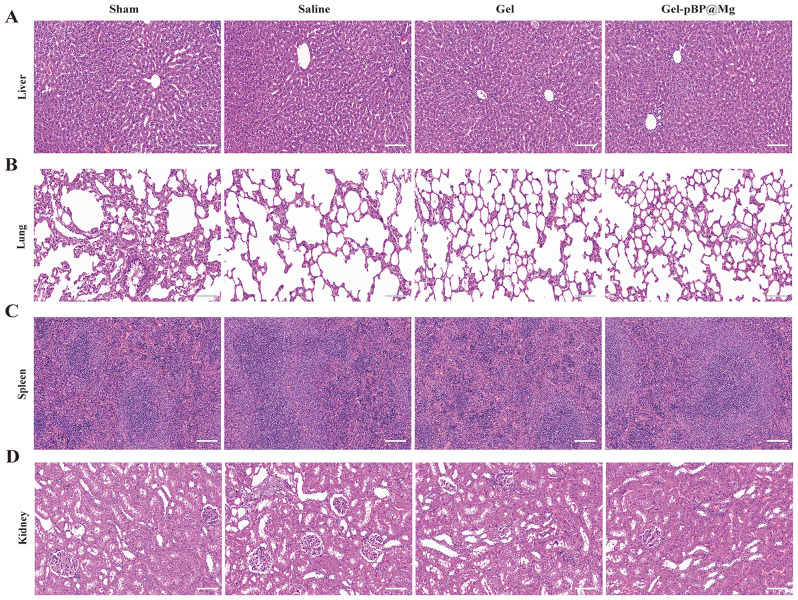
Systemic toxicity evaluation. HE staining of liver (**A**), lung (**B**), spleen (**C**), and kidney (**D**) in Sham, Saline, Gel and Gel-pBP@Mg groups at 28 days after modeling. Scale: 10 μm

The liver, lung, spleen, and kidney of experimental rats were collected for section and H&E staining 28 days after modeling **(**Fig. 9**)**. The results showed that compared with the sham group, there was no inflammatory infiltration in all tissues and organs of the saline group, Gel group, and Gel-pBP@Mg groups. The liver, lung, spleen, and kidney morphology were normal. This result suggests the high safety of local use of composite materials.

## Discussion

Here, we designed Gel-pBP@Mg, a drug delivery system for the effective treatment of myocardial infarction. The encapsulated pBP@Mg can undergo gradual degradation and subsequently release in the presence of elevated levels of ROS within the microenvironment of MI. The composite nanomaterial pBP@Mg integrates an anti-oxidative stress-inflammation reaction chain and pro-angiogenesis. The study showed that a local delivery system was effective and feasible in MI rats. Specifically, BPNSs inhibited oxidative stress-inflammation reaction chain and down-regulated NF-κB pathway to inhibit cardiomyocyte apoptosis. Mg loaded on the BPNSs surface can promote angiogenesis by activating the PI3K-Akt pathway. The nano hydrogel delivery system comprising composite materials improves heart function by mitigating MI-induced damage through complex interactions.

In MI individuals, excess ROS can cause cellular damage. However, BPNSs have strong anti-oxidative stress properties, and their degradation products are non-toxic to organisms. Several studies have investigated the osteogenic effects and nerve regeneration induction role of BPNSs in systemic diseases [11, 12, 44]. Moreover, BPNSs with a high specific surface area can more easily load drugs, metal ions, and miRNA [14, 35]. Clinical studies have found that early administration of Mg after the onset of MI may help in the treatment of MI. The benefit is especially high in patients who cannot be thrombolyzed [45]. This is similar to our animal model because the coronary arteries cannot be recanalized after suture ligation. Mg has been found to be essential for the normal physiologic function of vascular smooth muscle cells (VSMC), endothelial cells, and myocardium, and hypomagnesemia has been associated with increased cardiovascular morbidity and mortality. The protective effect of Mg on the myocardium is not only through pro-vascular effects, but also through anti-oxidative stress, antagonism of Ca^2+^, and reduction of heart rate and contractility [46]. Our study aimed to investigate using BPNSs to reduce oxidative stress-inflammation reaction chain and promote vascular regeneration in the infarction edge area by loading Mg onto the surface. The results showed a significant reduction in the damage to resident cardiomyocytes at the infarction site. Our data showed that BPNSs significantly inhibited oxidative stress-inflammation reaction chain and apoptosis in cardiomyocytes by down-regulating proteins related to the NF-κB signaling pathway, consistent with Weirun Li’s findings [41]. Previous studies have achieved a pro-angiogenic effect through the precise delivery of growth factors or cells, which is unconducive to clinical use [47, 48]. Herein, neovascularization in the infarction area was effectively achieved. In vivo and in vitro studies have confirmed that Mg promotes angiogenesis by activating the PI3K-Akt pathway. Notably, these in vivo experiments could convert M1 phenotypic macrophages into M2 phenotypic macrophages, which may be related to the role of Mg [49]. These data indicate that the synergistic effect of BPNSs and Mg improves the deterioration of the microenvironment in the infarction area, effectively inhibits the progression of infarction, and significantly improves cardiac function.

Recently, more research has tended to extend the life of biological materials through chemical modifications [10, 50]. The unique ability of BPNSs to resist oxidative stress brings some limitations. Water and oxygen present in the air can easily degrade BPNSs by oxidation. Herein, pBP@Mg coated with PDA showed a lower natural degradation rate, which aligns with Fan Wu [51]. MI is a disorder of local blood circulation; the injectable hydrogel material mimics the viscoelasticity of natural tissue and adapts to the shape of surrounding tissue, enabling local treatment in the infarct area [52]. Our research involved the development of an injectable hydrogel that is sensitive to ROS. The borate bond between Gel was broken by high ROS in the MI microenvironment, leading to a slow release of pBP@Mg. This controlled release method can continuously deliver pBP@Mg to the infarction site and play a lasting and accurate therapeutic effect. Additionally, the local administration will not cause damage to the surrounding healthy tissue, which is a good solution for the safety of biological materials. By modifying BPNSs and optimizing local delivery mode, Gel-pBP@Mg composite nano hydrogel has shown a significant therapeutic effect on improving cardiac function in vivo. Notably, in some results, Gel also showed the ability to scavenge ROS. To the best of our knowledge, many ROS-responsive hydrogels possess this ability. They have shown some ROS scavenging and anti-inflammatory response abilities in different disease models [53, 54]. This is due to the fact that in a ROS environment, chemical bond breaking in Gel consumes some of the ROS. He et al. obtained similar results using the same hydrogel in the treatment of diabetic wounds [31].

Several studies have increasingly emphasized the significance of biomaterials in cardiac tissue engineering. However, limited clinical trials have been conducted using injectable hydrogels for heart repair. This may be attributed to inadequate support from large animal studies. Yan Li studied the therapeutic effect of injectable materials through a pig MI model, which may provide data support for clinical transformation [55]. Additionally, the injection dose, foreign body reaction, and optimal treatment time window of composite nanohydrogels still need to be further determined before clinical use.

## Conclusion

We developed a novel composite nano hydrogel with excellent biocompatibility and can also design and achieve high ROS-responsive degradation characteristics according to the microenvironment characteristics in the infarction area post-MI. Based on this, the sustained release of composite nanomaterial pBP@Mg in the infarction area was realized to inhibit the oxidative stress-inflammation reaction chain, efficiently promote angiogenesis, improve microenvironment deterioration in the infarction area, and effectively inhibit infarction progression. In vitro studies have shown that the composite hydrogel system effectively achieved ROS clearance in CMs, maintained mitochondrial membrane potential anti-apoptosis, and promoted angiogenesis through the TRPM7/PI3k/Akt signaling pathway. Meanwhile, in vivo experiments showed that Gel-pBP@Mg can inhibit the occurrence of inflammatory storms in the infarction area by inhibiting NF-κB signaling pathway activation. Notably, at the site of infarction, Gel-pBP@Mg facilitated the M1 phenotypic macrophage differentiation into M2 phenotype, improving myocardial repair. Our new complex hydrogel, Gel-pBP@Mg, is more effective in utilizing the microenvironment characteristics of the infarction area to clear ROS and reduce the oxidative stress-inflammation reaction chain compared to previous studies. It promotes blood vessel formation in the infarction area, improves blood supply, alleviates myocardial injury, enhances myocardial function, and effectively inhibits adverse MI progression. Therefore, the effective treatment and inhibition of adverse events post-MI using Gel-pBP@Mg nano hydrogel is of utmost significance for the future clinical treatment and transformation of MI.

## Materials and methods

### Materials

Black phosphorus block was purchased from Alfa Aesar. Propylene carbonate, tetrabutylammonium hexafluorophosphate, magnesium chloride, polydopamine, N, N, N’, N ' -tetramethyl-1,3-propanediamine, 4(bromomethyl)benzeneboronic acid, dimethylformamide, tetrahydrofuran, and polyvinyl alcohol were purchased from Macklin. Hydrogen peroxide (H_2_O_2_) solution (30%) was purchased from China Beijing Chemical Industry Co. Anti-Bax (ab32503), anti-Bcl-2 (ab194583), anti-caspase-3 (ab184787), GAPDH (ab8245), anti-β-actinin (ab8227) were purchased from Abcam, USA. anti-PI3K (T40064), anti-p-PI3K (T40065), anti-AKT (T55561), anti-p-AKT (T40067), anti-TNF-α (PY19810), anti-IL-6 (TD6087), anti-IL-10 (TD6894), anti-CD206 (TD4149), anti-NF-κB (PU949015), anti-p-IκBa (TP70389), affiniPure goat anti-mouse lgG (M21001) and affiniPure goat anti-rabbit lgG (M21002) were purchased from Abmart. 2,2-Diphenyl-1-picrylhydrazyl (DPPH) was provided by Energy Chemical (China). 2, 2’-azino-bis(3-ethylbenzothiazoline-6-sulfonic acid) (ABTS) and CCK-8 solution was provided by Solarbio (China). MMP (JC-1) assay kit and reactive oxygen species assay kit were purchased from Beyotime (China). H9C2, RAW264.7, and human umbilical vein endothelial cells (HUVECs) were supplied by the Cell Bank of the Chinese Academy of Sciences. DMEM high glucose base medium, fetal bovine serum and Penicillin-Streptomycin were purchased from Servicebio (Wuhan, China). HUVEC cell specific medium was purchased from Procell Life Science & Technology Co., Ltd. (CM-0122, Wuhan, China). RIPA Lysis Buffer, BCA protein quantitative kit, polyvinylidene fluoride film and ECL hypersensitive luminescent solution were purchased from Servicebio (Wuhan, China).

### Preparation of BPNSs and pBP@Mg

BP blocks were synthesized by a cubic press with six alloy mallets under high temperature and pressure. The black phosphorus block precursor was pre-pressed at room temperature and then rapidly heated to 1000 °C for 10 min; large chunks of BP formed after cooling and releasing pressure.

BPNSs were then prepared by electrochemical stripping: BP crystal as the cathode and platinum plate as the anode. In propylene carbonate (PC) and tetrabutylammonium hexafluorophosphate at a constant voltage of − 5 V. The solution was collected into a centrifuge tube, and the stripped BP tablets were dispersed into anhydrous acetone 1 h after ultrasound, followed by centrifugation at 1500 rpm for 10 min to remove thick BP tablets. Next, the BPNSs were collected by being centrifuged at 5000 rpm for 10 min. After removing the supernatant, BPNSs were dispersed into ultra-pure water.

Subsequently, 7.5 mg of MgCl was added to BPNSs suspension (0.5 mg mL^–1^), ultrasonic for 3 min, and stirred away from light for 4 h. The mixture was centrifuged at 5000 rpm for 15 min. BP@Mg was collected and rinsed in ultra-pure water. Then, BP@Mg was added to an alkaline solution with pH = 8.5, and 100 µL of dopamine hydrochloride aqueous solution (50 mg mL^− 1^) was added, stirred in the dark for 5 h, and centrifuged at 5000 rpm for 15 min to obtain pBP@Mg.

### Preparation of ROS-responsive hydrogels

According to previous literature [30], N, N, N ‘, N ‘-tetramethyl-1, 3-propylenediamine (0.1 g, 0.75 mmol), and 4-(bromomethyl) phenylboric acid (0.5 g, 2.3 mmol) were dissolved in dimethylformamide (10 mL) and mixed, respectively. After stirring at 60 °C overnight, tetrahydrofuran (THF) was poured (100 mL), filtered, and washed with THF (3 × 20 mL), followed by drying overnight under a vacuum to obtain pure hydrogel precursor. Next, they were dissolved in deionized water at 25 °C, while PVA (31,145 kDa; 98% hydrolyzed) was dissolved in deionized water at 90 °C. Equal volumes of hydrogel precursor and PVA solutions were mixed by vigorous agitation to obtain a uniform hydrogel.

### Characterization of BPNSs and pBP@Mg

Morphology and surface elements were scanned using transmission electron microscopy (JEOL, JEM-F200, Japan) at an accelerated voltage of 200 kV. The thickness of nanomaterials was measured by atomic force microscopy (Bruker, Dimension Icon, Germany). XRD (X’Pert PRO MPD) was used to detect BPN crystallinity. The characteristic absorption peaks were detected by XPS (Thermo, Scientific K-Alpha, USA). The fine spectra included C, Mg, P, and N. Raman spectroscopy was performed by Raman microscopy equipment (Horiba LabRAM HR Evolution, Japan) under laser excitation at 532 nm. Fourier infrared spectroscopy (Nicolet iS 10) detected chemical bond information in the 500–4000 cm^–1^ wave number range. The Zeta potential (Malvern Zetasizer Nano ZS90) was used to detect the BPNSs, BP@PDA, and pBP@Mg potentials.

### Stability of BPNSs and pBP@Mg

BPNSs and pBP@Mg were dispersed in ultra-pure water (100 µg mL^–1^) and exposed to air. Their light absorption properties were studied on days 1, 4, 7, 14, 21, and 28 using a UV-VIS spectrometer (3600 UV-VIS spectrometer, Shimadzu, Japan).

### Characterization of gel, Gel-BP, and Gel-pBP@Mg

The microstructures of hydrogels were observed by scanning electron microscopy (ZEISS, GeminiSEM 300, Germany). The hydrogels were frozen at − 80 °C and then freeze-dried to remove the water. The cross-section of the hydrogel sample is then coated with gold to improve its electrical conductivity.

### Rheological Testing

The modulus was tested using a rheometer (Haake Mars60, Germany). The storage modulus G ‘and the loss modulus G “are obtained using a fixed strain of 0.5% on a 20 mm parallel plate at room temperature; the frequency range was 0.1–10 Hz.

### Resistivity experiment

The resistivity of hydrogel was measured by an ST2242 AC four-probe tester (KDY-1; Guangzhou Kunde Technology Co.Ltd. China). The probe was inserted into 4 mm thick and 30 mm diameter Gel-pBP@Mg and tested resistivity in parallel.

### Swelling test

The swelling property of hydrogel was measured by the equilibrium swelling ratio (SR). The initial weight of hydrogel W0 was measured. The hydrogel was then immersed in PBS at 37 °C. Subsequently, it was removed after 24 h and gently wiped with filter paper. The weight W_t_ was measured again, and the swelling ratio of hydrogel was calculated by the formula:

$$Swelling ratio (g/g)=({W}_{t}-{W}_{0})/{W}_{0}$$

W_t_ is the mass after soaking for 24 h, and W_0_ is the initial mass before measurement.

### Slow-release assay

The release of elemental phosphorus from degradable BPNSs in the Gel–pBP@Mg hydrogel scaffold was analyzed using inductively coupled plasma atomic emission spectroscopy (ICP-AES). Each hydrogel sample (0.5 ml) was immersed in 6 ml of normal PBS and incubated at 37 °C for up to 21 days.

### ROS scavenging ability of hydrogels

To test the ROS response of hydrogel, two sample vials were filled with 2 mL of ultra-pure water, and H_2_O_2_ (10 mM) and ROS-responsive hydrogel (0.5 mL) were added. The hydrogel decomposition was observed after being placed at 37 °C for 2 h.

The antioxidant activity of composite hydrogel was evaluated by scavenging DPPH free radicals. The DPPH solution of 0.04 mg mL^–1^ was prepared with ethanol as solvent. Then, 500 µL of DPPH solution was added to different hydrogel surfaces. In the blank group, only the DPPH solution was added and left at room temperature for 2 h in darkness. The absorbance of supernatant at 517 nm was measured by UV-VIS spectrophotometer. The ability of the hydrogel to scavenge DPPH free radicals was calculated by the equation:

$$Scavenging effect=\frac{{A}_{control}-{A}_{sample}}{{A}_{control}}\times 100\%$$

The ABTS method was performed to determine the total antioxidant capacity of composite hydrogels. The ABTS test solution was prepared according to the instructions. ABTS detection solution of 500 µL was added to the surface of different hydrogels, mixed well, and left to stand at room temperature for 6 min away from light. In the blank group, only the ABTS detection solution was added. The absorbance at 405 nm was measured. The free radical scavenging rate of hydrogel ABTS was calculated by the equation:

$$Scavenging effect=\frac{{A}_{control}-{A}_{sample}}{{A}_{control}}\times 100\%$$

### In vitro cell culture

H9C2 and RAW264.7 cells were cultured in medium containing 89% DMEM high glucose base medium, 10% fetal bovine serum and 1% Penicillin-Streptomycin in a 5% CO_2_ incubator at 37 °C. HUVEC cells were cultured in special medium in a 5% CO_2_ incubator at 37 °C.

### Cell viability and biosafety assay

CMs were inoculated on composite hydrogels of different concentrations at a density of 5000 cells per well in a 96-well culture plate. When the cells reached 70–80% confluent, 0.1mM of H_2_O_2_ was added and treated for 24 h. Consequently, 10 µL of CCK-8 solution was added to each well and treated for 2 h. The absorbance was measured at 450 nm wavelength, and the number of living cells was measured by drawing a standard curve according to the OD value.

### Determination of living/dead cell viability

The cell viability was evaluated qualitatively by calcein staining. After inoculating cells on hydrogels containing different composites for 12 h, calcein by 10% cell medium volume was added, incubated at 37 °C for 30 min, and washed twice with PBS. The cells were observed by fluorescence microscopy (Axio Vert A1, ZEISS, 10×/0.32).

### Evaluation of reactive oxygen generation and clearance

The DCFH-DA was used to detect intracellular superoxide anions and various ROS levels. CMs were inoculated on hydrogels containing different composite materials. Treatment with 0.1 mM of H_2_O_2_ for 12 h induced oxidative stress. Then DCFH-DA was added, incubated at 37 °C for 30 min in the dark, and was observed under a fluorescence microscope (Axio Vert A1, ZEISS, 10×/0.32). The relative fluorescence intensity of DCFH-DA was calculated using Image-Pro Plus software.

### Mitochondrial membrane potential detection

The CMs were inoculated on the hydrogel for 12 h and treated with 0.1 mM H_2_O_2_ for 12 h. The JC-1 detection solution was added in darkness, incubated at 37 °C for 20 min, washed twice with 1× buffer, and observed under a fluorescence microscope (Axio Vert A1, ZEISS, 40×/0.85).

### Apoptosis flow cytometry

The CMs were inoculated on the hydrogel for 12 h and treated with 0.1 mM H_2_O_2_ for 12 h. The Annexin V-FITC Apoptosis Detection Kit (Beyotime, China) was used to detect apoptosis levels. Cells were collected by trypsinization without EDTA and washed twice with PBS. Cells were collected into centrifuge tubes and incubated at room temperature for 20 min with a detection reagent. Flow cytometry was then performed.

### In vitro migration and tube formation of HUVEC

The back of a 6-well plate was marked every 0.5 cm. HUVEC was cultured in 6-well plates with a cell density of 6 × 10^5^ cells. When the cells were fully fused, a 10 µL pipette tip was used to scratch along the center. Then, it was washed with 1×PBS twice to remove the shed cells. HUVEC was then treated with a medium containing treatment factor. The migration was observed 24 h later with a fluorescence microscope (Axio Vert A1, ZEISS, 10×/0.32).

Subsequently, 50 µL of matrix glue was added to a 96-well plate and incubated at 37 °C for 1 h. HUVECs were added at a density of 3 × 10^4^ cells per well and treated separately with a solution containing the treatment factor. Finally, HUVECs were incubated for 6 h, and the tube formation was observed by fluorescence microscopy (Axio Vert A1, ZEISS, 10×/0.32).

### MI and hydrogel injection model in rats

Male SD rats (aged seven weeks; 240–260 g) were sourced from Vital River (Beijing, China). The rats were mechanically ventilated with a ventilator and anesthetized with 10% chloral hydrate. The left lateral thoracotomy exposed the heart and induced MI by ligation of the anterior descending branch of the left coronary artery with 8 − 0 polypropylene sutures. The ligation was maintained during subsequent experiments. Myocardial ischemia was determined by regional cyanosis on the front of the heart [41]. The hydrogel (20 µL each) was injected into five sites in and around the infarct area. Animal experimental procedures were approved by the Institutional Animal Care and Use Committee, Huazhong University of Science and Technology (IACUC Number: 3440).

### Electrocardiogram and echocardiogram in rats

The Vevo 2100 ultrasound imaging system was employed to evaluate the left ventricular function. The MI model was established for 24 h, the first ultrasound and electrocardiogram were performed, and a second test was performed on 28 days. The horizontal placement of the papillary muscle was assessed using a short axis view, guided by two-dimensional chest ultrasound. This technique was employed to determine parameters such as EF, FS, LVIDs, and LVIDd. All measurements were averaged over three consecutive heart cycles.

### Histomorphological analysis

The rats were euthanized 28 days after surgery. The heart was quickly removed and fixed in paraformaldehyde. The heart was paraffin-embedded, sliced from apex to ligation, and stained with Masson (Servicebio, Wuhan, China). The infarct size and wall thickness of the MI area were measured. H&E (Servicebio, Wuhan, China) staining was used to observe the histological morphology of the MI area and the toxicity of other organs.

### Immunofluorescence detection

DHE immunofluorescence staining was performed on the frozen sections to evaluate ROS levels in the infarct area. Images were obtained using fluorescence microscopy (Axio Vert A1, ZEISS, 40×/0.85). Tunel and Caspase-3 staining were used to evaluate the apoptosis of cardiomyocytes. The number of new vessels was detected by α-SMA and CD31 immunofluorescence co-localization. The paraffin sections were stained with TNF-α, iNOS, CD206, and IL-10 immunofluorescence.

### WB analysis

CMs, HUVEC and RAW264.7 were washed twice using PBS and added RIPA Lysis Buffer for 30 min, centrifuged at 13,400 g for 10 min, and cell supernatant was collected. The protein extracted from the heart tissue was first ground with a grinding bead and then broken by ultrasound for five cycles. The supernatant was collected after centrifugation. After measuring the protein concentration with a BCA protein quantitative kit, a loading buffer was added, boiled, and stored at − 20 °C. Electrophoresis was performed using SDS-PAGE and subsequently transferred to polyvinylidene fluoride film. Then, it was incubated with the blocking solution, primary antibody, and secondary antibody successively. Incubated with ECL hypersensitive luminescent solution and imaged with chemiluminescence apparatus.

### RNA sequencing, differential expression gene identification, cluster analysis, and functional enrichment analysis

Fresh heart tissues of the normal saline group and the Gel-pBP@Mg group were obtained 28 days after surgery for RNA-seq analysis, with three samples in each group. Total RNA was extracted by TRIzol reagent according to the instructions. RNA purity and quantification were identified using the NanoDrop 2000 spectrophotometer (Thermo Scientific, USA), and RNA integrity was assessed using Agilent 2100 Bioanalyzer (Agilent Technologies, Santa Clara, CA, USA). Transcriptome libraries were constructed according to the instructions using the VAHTS Universal V5 RNA-seq Library Prep kit. Illumina Novaseq 6000 sequencing platform was used to sequence the library, and 150 bp double-ended reads were generated. DESeq2 software was used to analyze the differentially expressed genes. Subsequently, GO and KEGG enrichment analysis of differentially expressed genes was performed based on a hypergeometric distribution algorithm. R (v 3.2.0) was used to plot the TOP five items of significant enrichment functions.

### Statistical analysis

All experiments were performed on at least three individual samples. All data were expressed as mean ± standard deviation. The Shapiro–Wilk Normality test and one-way ANOVA was performed using GraphPad Prism 8.0. Statistical significance was defined as *p<0.05;**p<0.01;***p<0.001;****p<0.0001.

### Electronic supplementary material

Below is the link to the electronic supplementary material.

Supplementary Material 1

Supplementary Material 2

## Data Availability

No datasets were generated or analysed during the current study.

## References

[1] Kalogeris T, Baines CP, Krenz M, Korthuis RJ. CELL BIOLOGY OF ISCHEMIA/REPERFUSION INJURY, in: K.W. Jeon, editor International Review of Cell and Molecular Biology, Vol 298, 2012, pp. 229–317. 10.1016/B978-0-12-394309-5.00006-7 PMC3904795 22878108

[2] Sack MN, Fyhrquist FY, Saijonmaa OJ, Fuster V, Kovacic JC. Basic Biology of oxidative stress and the Cardiovascular System: part 1 of a 3-Part series. J Am Coll Cardiol. 2017;70:196–211. 28683968 10.1016/j.jacc.2017.05.034 PMC5551687

[3] Yalta K, Yilmaz MB, Yalta T, Palabiyik O, Taylan G, Zorkun C. Late Versus Early myocardial remodeling after Acute myocardial infarction: a comparative review on mechanistic insights and clinical implications. J Cardiovasc Pharmacol Therap. 2020;25:15–26. 31416353 10.1177/1074248419869618

[4] Nahrendorf M, Pittet MJ, Swirski FK. Monocytes: protagonists of infarct inflammation and repair after myocardial infarction. Circulation. 2010;121:2437–45. 20530020 10.1161/CIRCULATIONAHA.109.916346 PMC2892474

[5] W.H.O Cardiovascular diseases (CVDs). W.H.O Cardiovascular diseases (CVDs). https://www.who.int/news-room/fact-sheets/detail/cardiovascular-diseases-(cvds). (Accessed Oct 2022.).

[6] Mitsis A, Gragnano F. Myocardial infarction with and without ST-segment elevation: a contemporary reappraisal of similarities and differences, current cardiology reviews, 17 (2021) e230421189013. 10.2174/1573403X16999201210195702 PMC8762150 33305709

[7] Gong FF, Vaitenas I, Malaisrie SC, Maganti K. Mechanical complications of Acute myocardial infarction: a review. JAMA Cardiol. 2021;6:341–9. 33295949 10.1001/jamacardio.2020.3690

[8] Azzalini L, Karmpaliotis D, Santiago R, Mashayekhi K, Di Mario C, Rinfret S, Nicholson WJ, Carlino M, Yamane M, Tsuchikane E, Brilakis ES. Contemporary issues in chronic total occlusion percutaneous coronary intervention. JACC: Cardiovasc Interventions. 2022;15:1–21. 10.1016/j.jcin.2021.09.027 34991814

[9] Ling X, Wang H, Huang S, Xia F, Dresselhaus MS. The renaissance of black phosphorus. Proc Natl Acad Sci USA. 2015;112:4523–30. 25820173 10.1073/pnas.1416581112 PMC4403146

[10] Zeng G, Chen Y. Surface modification of black phosphorus-based nanomaterials in biomedical applications: strategies and recent advances. Acta Biomater. 2020;118:1–17. 33038527 10.1016/j.actbio.2020.10.004

[11] Xu Y, Xu C, He L, Zhou J, Chen T, Ouyang L, Guo X, Qu Y, Luo Z, Duan D. Stratified-structural hydrogel incorporated with magnesium-ion-modified black phosphorus nanosheets for promoting neuro-vascularized bone regeneration. Bioactive Mater. 2022;16:271–84. 10.1016/j.bioactmat.2022.02.024 PMC8965728 35386320

[12] Qing Y, Li R, Li S, Li Y, Wang X, Qin Y. Advanced Black Phosphorus nanomaterials for Bone Regeneration. Int J Nanomed. 2020;15:2045–58. 10.2147/IJN.S246336 PMC7104107 32273701

[13] Huang Y, Qiao J, He K, Bliznakov S, Sutter E, Chen X, Luo D, Meng F, Su D, Decker J, Ji W, Ruoff RS, Sutter P. Interact Black Phosphorus Oxygen Water Chem Mater. 2016;28:8330–9.

[14] Zeng X, Luo M, Liu G, Wang X, Tao W, Lin Y, Ji X, Nie L, Mei L. Polydopamine-modified black Phosphorous Nanocapsule with enhanced Stability and Photothermal Performance for Tumor Multimodal treatments. Adv Sci (Weinheim Baden-Wurttemberg Germany). 2018;5:1800510. 10.1002/advs.201800510 PMC6193171 30356942

[15] Lee H, Dellatore SM, Miller WM, Messersmith PB. Mussel-inspired Surface Chemistry for Multifunctional Coatings. Sci (New York N Y). 2007;318:426–30. 10.1126/science.1147241 PMC2601629 17947576

[16] Shih H, Lee B, Lee RJ, Boyle AJ. The Aging Heart and Post-infarction Left ventricular remodeling. J Am Coll Cardiol. 2011;57:9–17. 21185495 10.1016/j.jacc.2010.08.623 PMC3031493

[17] Whelan RS, Kaplinskiy V, Kitsis RN. Cell Death Pathogenesis Heart Disease: Mech Significance. 2010;72:19–44. 10.1146/annurev.physiol.010908.163111 PMC12973270 20148665

[18] Liu M, Wang R, Liu J, Zhang W, Liu Z, Lou X, Nie H, Wang H, Mo X, Abd-Elhamid AI, Zheng R, Wu J. Incorporation of magnesium oxide nanoparticles into electrospun membranes improves pro-angiogenic activity and promotes diabetic wound healing. Biomaterials Adv. 2022;133:112609. 10.1016/j.msec.2021.112609 35525752

[19] Xu L, Willumeit-Römer R, Luthringer-Feyerabend BJC. Effect of magnesium-degradation products and hypoxia on the angiogenesis of human umbilical vein endothelial cells. Acta Biomater. 2019;98:269–83. 30794987 10.1016/j.actbio.2019.02.018

[20] Sreenivasamurthy SA, Akhter FF, Akhter A, Su Y, Zhu D. Cellular mechanisms of biodegradable zinc and magnesium materials on promoting angiogenesis. Biomaterials Adv. 2022;139:213023. 10.1016/j.bioadv.2022.213023 35882117

[21] Liu M, Wang X, Cui J, Wang H, Sun B, Zhang J, Rolauffs B, Shafiq M, Mo X, Zhu Z, Wu J. Electrospun flexible magnesium-doped silica bioactive glass nanofiber membranes with anti-inflammatory and pro-angiogenic effects for infected wounds. J Mater Chem B. 2023;11:359–76. 36507933 10.1039/D2TB02002E

[22] Nasr SM, Rabiee N, Hajebi S, Ahmadi S, Fatahi Y, Hosseini M, Bagherzadeh M, Ghadiri AM, Rabiee M, Jajarmi V, Webster TJ. Biodegradable nanopolymers in Cardiac tissue Engineering: from Concept towards Nanomedicine. Int J Nanomed. 2020;15:4205–24. 10.2147/IJN.S245936 PMC7314574 32606673

[23] Kc P, Hong Y, Zhang G. Cardiac tissue-derived extracellular matrix scaffolds for myocardial repair: advantages and challenges. Regenerative Biomaterials. 2019;6:185–99. 31404421 10.1093/rb/rbz017 PMC6683951

[24] Bernhard S, Tibbitt MW. Supramolecular engineering of hydrogels for drug delivery. Adv Drug Deliv Rev. 2021;171:240–56. 33561451 10.1016/j.addr.2021.02.002

[25] Rufaihah AJ, Seliktar D. Hydrogels for therapeutic cardiovascular angiogenesis. Adv Drug Deliv Rev. 2016;96:31–9. 26212158 10.1016/j.addr.2015.07.003

[26] Bejleri D, Davis ME. Decellularized extracellular matrix materials for Cardiac Repair and Regeneration. Adv Healthc Mater. 2019;8:e1801217. 30714354 10.1002/adhm.201801217 PMC7654553

[27] Zhang Y, Mu W, Zhang Y, He X, Wang Y, Ma H, Zhu T, Li A, Hou Q, Yang W, Ding Y, Ramakrishna S, Li H. Recent advances in Cardiac patches: materials, preparations, and Properties. Volume 8. ACS biomaterials science & engineering; 2022. pp. 3659–75. 10.1021/acsbiomaterials.2c00348 36037313

[28] Wang L, Yu Y, Zhao X, Zhang Z, Yuan X, Cao J, Meng W, Ye L, Lin W, Wang G. A biocompatible self-powered piezoelectric poly(vinyl alcohol)-Based hydrogel for Diabetic Wound Repair. Volume 14. ACS applied materials & interfaces; 2022. pp. 46273–89. 10.1021/acsami.2c13026 36195572

[29] D. Li, K. Chen, H. Tang, S. Hu, L. Xin, X. Jing, Q. He, S. Wang, J. Song, L. Mei, R.D. Cannon, P. Ji, H. Wang, T. Chen. A logic-based diagnostic and therapeutic hydrogel with Multistimuli responsiveness to Orchestrate Diabetic Bone Regeneration. Adv Mater (Deerfield Beach Fla). 2022;34:e2108430. 10.1002/adma.202108430 34921569

[30] Wang C, Wang J, Zhang X, Yu S, Wen D, Hu Q, Ye Y, Bomba H, Hu X, Liu Z, Dotti G, Gu Z. In situ formed reactive oxygen species-responsive scaffold with gemcitabine and checkpoint inhibitor for combination therapy. Sci Transl Med, 10 (2018). 10.1126/scitranslmed.aan3682 29467299

[31] Zhao H, Huang J, Li Y, Lv X, Zhou H, Wang H, Xu Y, Wang C, Wang J, Liu Z. ROS-scavenging hydrogel to promote healing of bacteria infected diabetic wounds. Biomaterials. 2020;258:120286. 32798744 10.1016/j.biomaterials.2020.120286

[32] Zheng Z, Lei C, Liu H, Jiang M, Zhou Z, Zhao Y, Yu CY, Wei H. A ROS-Responsive liposomal composite hydrogel integrating improved mitochondrial function and pro-angiogenesis for efficient treatment of myocardial infarction. Adv Healthc Mater. 2022;11:e2200990. 35848825 10.1002/adhm.202200990

[33] Hu K, Xie L, Zhang Y, Hanyu M, Yang Z, Nagatsu K, Suzuki H, Ouyang J, Ji X, Wei J, Xu H, Farokhzad OC, Liang SH, Wang L, Tao W, Zhang MR. Marriage of black phosphorus and Cu(2+) as effective photothermal agents for PET-guided combination cancer therapy. Nat Commun. 2020;11:2778. 32513979 10.1038/s41467-020-16513-0 PMC7280494

[34] Harrington MJ, Masic A, Holten-Andersen N, Waite JH, Fratzl P. Iron-clad fibers: a metal-based biological strategy for hard flexible coatings. Sci (New York N Y). 2010;328:216–20. 10.1126/science.1181044 PMC3087814 20203014

[35] Jiang H, Xia Q, Zheng J, Bu J, Li R, Cai Z, Ling K. Mn(2+) modified black phosphorus nanosheets with enhanced DNA adsorption and affinity for robust sensing. Biosens Bioelectron. 2022;216:114622. 35973273 10.1016/j.bios.2022.114622

[36] Yang G, Wan X, Gu Z, Zeng X, Tang J. Near infrared photothermal-responsive poly(vinyl alcohol)/black phosphorus composite hydrogels with excellent on-demand drug release capacity. J Mater Chem B. 2018;6:1622–32. 32254278 10.1039/C7TB03090H

[37] Kornfeld OS, Hwang S, Disatnik MH, Chen CH, Qvit N, Mochly-Rosen D. Mitochondrial reactive oxygen species at the heart of the matter: new therapeutic approaches for cardiovascular diseases. Circul Res. 2015;116:1783–99. 10.1161/CIRCRESAHA.116.305432 PMC4443500 25999419

[38] Wang W, Wong NK, Bok SL, Xu Y, Guo Y, Xu L, Zuo M, St Croix CM, Mao G, Kapralov A, Bayir H, Kagan VE, Yang D. Visualizing Cardiolipin in situ with HKCL-1 M, a highly selective and sensitive fluorescent probe. J Am Chem Soc. 2023;145:11311–22. 37103240 10.1021/jacs.3c00243 PMC10214440

[39] Ryazanova LV, Rondon LJ, Zierler S, Hu Z, Galli J, Yamaguchi TP, Mazur A, Fleig A, Ryazanov AG. TRPM7 is essential for mg(2+) homeostasis in mammals. Nat Commun. 2010;1:109. 21045827 10.1038/ncomms1108 PMC3060619

[40] Zhang X, Zu H, Zhao D, Yang K, Tian S, Yu X, Lu F, Liu B, Yu X, Wang B, Wang W, Huang S, Wang Y, Wang Z, Zhang Z. Ion channel functional protein kinase TRPM7 regulates mg ions to promote the osteoinduction of human osteoblast via PI3K pathway: in vitro simulation of the bone-repairing effect of Mg-based alloy implant. Acta Biomater. 2017;63:369–82. 28882757 10.1016/j.actbio.2017.08.051

[41] Li W, Chen P, Pan Y, Lu L, Ning X, Liu J, Wei J, Chen M, Zhao P, Ou C. Construction of a Band-Aid like Cardiac Patch for myocardial infarction with controllable H(2) S release, Advanced science (Weinheim, Baden-Wurttemberg. Germany). 2022;9:e2204509. 10.1002/advs.202204509 PMC9762300 36285675

[42] Vazir A, Fox K, Westaby J, Evans MJ, Westaby S. Can we remove scar and fibrosis from adult human myocardium? Eur Heart J. 2019;40:960–6. 30203057 10.1093/eurheartj/ehy503

[43] Frangogiannis NG. Pathophysiology of myocardial infarction. Compr Physiol. 2015;5:1841–75. 26426469 10.1002/cphy.c150006

[44] Xu C, Xu Y, Yang M, Chang Y, Nie A, Liu Z, Wang J, Luo Z. Black-Phosphorus-Incorporated Hydrogel as a conductive and biodegradable platform for enhancement of the neural differentiation of mesenchymal stem cells, 30 (2020) 2000177.

[45] Antman EM. Magnesium in acute myocardial infarction: overview of available evidence. Am Heart J. 1996;132:487–95. 8694009 10.1016/S0002-8703(96)90341-5

[46] Kolte D, Vijayaraghavan K, Khera S, Sica DA, Frishman WH. Role of Magnesium in Cardiovascular diseases. Cardiol Rev, 22 (2014). 10.1097/CRD.0000000000000003 24896250

[47] Koike N, Fukumura D, Gralla O, Au P, Schechner JS, Jain RK. Tissue engineering: creation of long-lasting blood vessels. Nature. 2004;428:138–9. 15014486 10.1038/428138a

[48] Lin YD, Luo CY, Hu YN, Yeh ML, Hsueh YC, Chang MY, Tsai DC, Wang JN, Tang MJ, Wei EI, Springer ML, Hsieh PC. Instructive nanofiber scaffolds with VEGF create a microenvironment for arteriogenesis and cardiac repair. Sci Transl Med. 2012;4:146ra109. 22875829 10.1126/scitranslmed.3003841

[49] Saifullah B, Arulselvan P, El Zowalaty ME, Tan WS, Fakurazi S, Webster TJ, Baby R, Hussein MZ. A novel para-amino salicylic acid Magnesium Layered Hydroxide Nanocomposite Anti-tuberculosis Drug Delivery System with enhanced in vitro therapeutic and anti-inflammatory properties. Int J Nanomed. 2021;16:7035–50. 10.2147/IJN.S297040 PMC8526802 34703226

[50] Zhang Y, Ma C, Xie J, Ågren H, Zhang H. Black Phosphorus/Polymers: Status and challenges. Adv Mater (Deerfield Beach Fla). 2021;33:e2100113. 10.1002/adma.202100113 34323318

[51] Wu F, Zhang M, Chu X, Zhang Q, Su Y, Sun B, Lu T, Zhou N, Zhang J, Wang J, Yi X. Black phosphorus nanosheets-based nanocarriers for enhancing chemotherapy drug sensitiveness via depleting mutant p53 and resistant cancer multimodal therapy. Chem Eng J. 2019;370:387–99. 10.1016/j.cej.2019.03.228

[52] Zhang L, Li T, Yu Y, Shi K, Bei Z, Qian Y, Qian Z. An injectable conductive hydrogel restores electrical transmission at myocardial infarct site to preserve cardiac function and enhance repair. Bioactive Mater. 2023;20:339–54. 10.1016/j.bioactmat.2022.06.001 PMC9210214 35784639

[53] Zhang X, Sun Y, Yang R, Liu B, Liu Y, Yang J, Liu W. An injectable mitochondria-targeted nanodrug loaded-hydrogel for restoring mitochondrial function and hierarchically attenuating oxidative stress to reduce myocardial ischemia-reperfusion injury. Biomaterials. 2022;287:121656. 35792386 10.1016/j.biomaterials.2022.121656

[54] Huang L, Wang J, Kong L, Wang X, Li Q, Zhang L, Shi J, Duan J, Mu H. ROS-responsive hyaluronic acid hydrogel for targeted delivery of probiotics to relieve colitis. Int J Biol Macromol. 2022;222:1476–86. 36195227 10.1016/j.ijbiomac.2022.09.247

[55] Li Y, Chen X, Jin R, Chen L, Dang M, Cao H, Dong Y, Cai B, Bai G, Gooding JJ, Liu S, Zou D, Zhang Z, Yang C. Injectable hydrogel with MSNs/microRNA-21-5p delivery enables both immunomodification and enhanced angiogenesis for myocardial infarction therapy in pigs. Sci Adv, 7 (2021). 10.1126/sciadv.abd6740 PMC7904259 33627421

