# Supplementary material for: Targeted delivery of black phosphorus nanosheets by ROS responsive complex hydrogel based on angiogenesis and antioxidant promotes myocardial infarction repair
Source: J Nanobiotechnology. 2024 Jul 22;22:433. doi: 10.1186/s12951-024-02685-0 (PMC11265071; doi:10.1186/s12951-024-02685-0)
Supplement: Supplementary file 2 — Supplementary Material 2 [file 12951_2024_2685_MOESM2_ESM.docx]

Supplementary material

**Title:** Targeted delivery of black phosphorus nanosheets by ROS responsive complex hydrogel based on angiogenesis and antioxidant promotes myocardial infarction repair

*Jiahui Zhang^1^, Di Sun^1^, Yishan Guo^1^, Junran Tong, Qingyi Liu, Ran Gao*, Yumiao Wei* and Xiaopeng Guo**


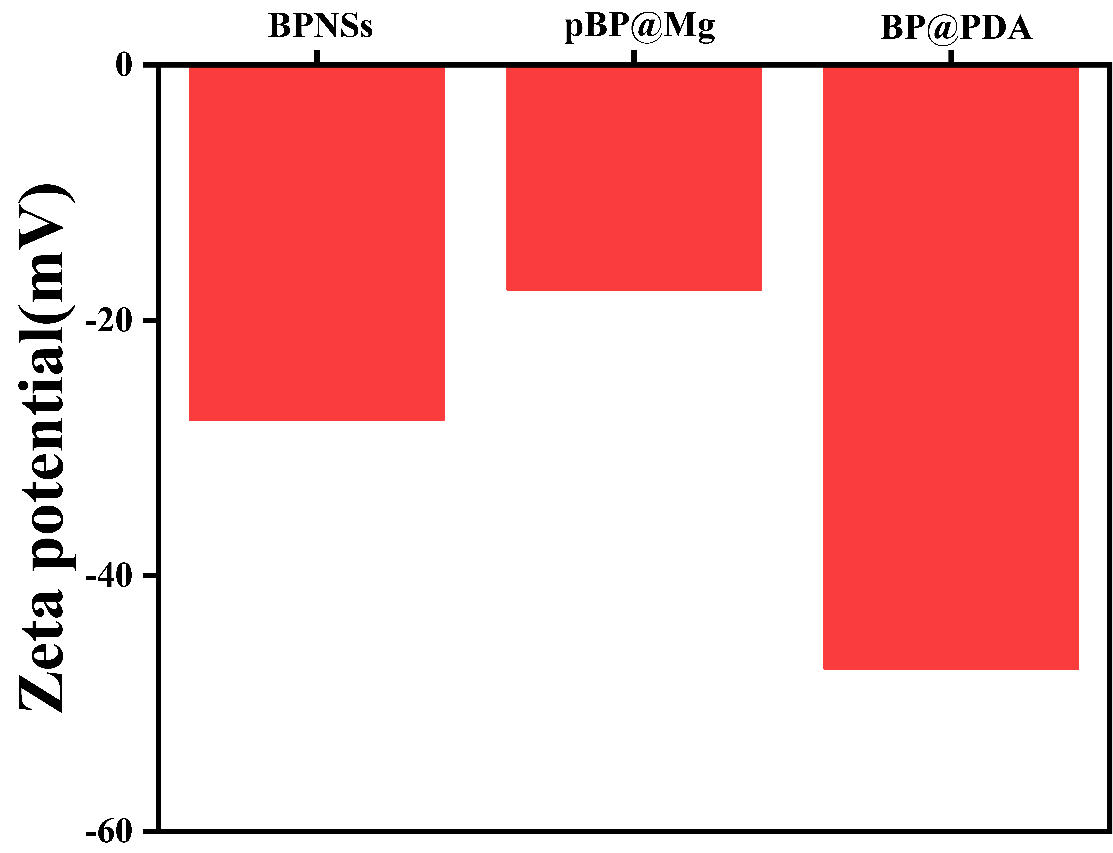


**Figure S1.** Zeta potentials for BPNSs, pBP@Mg and BP@PDA.


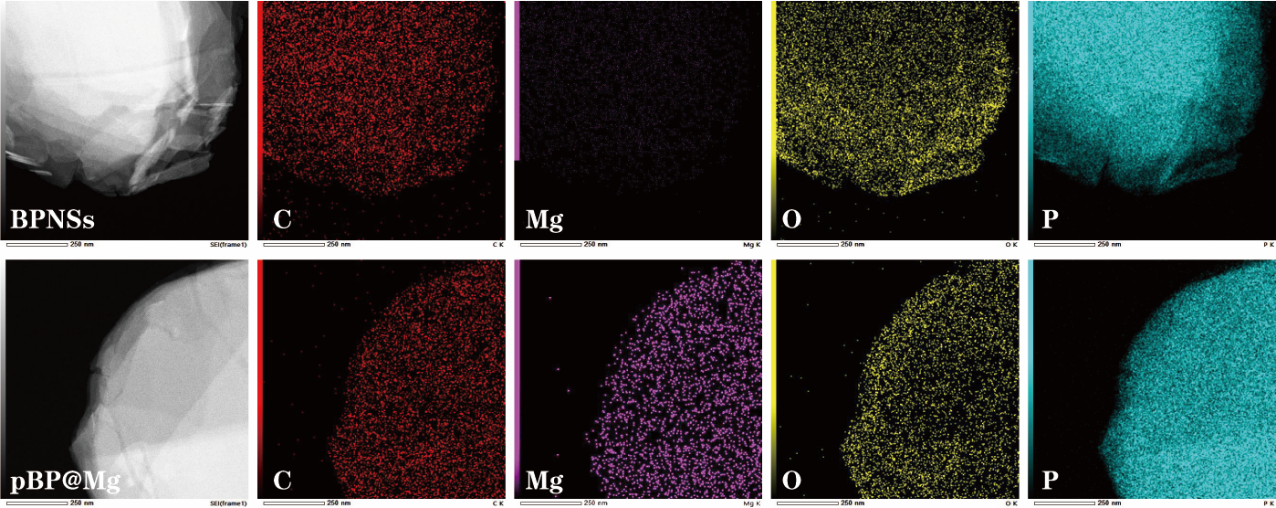


**Figure S2.** TEM-EDS elemental mapping images of BPNSs and pBP@Mg.


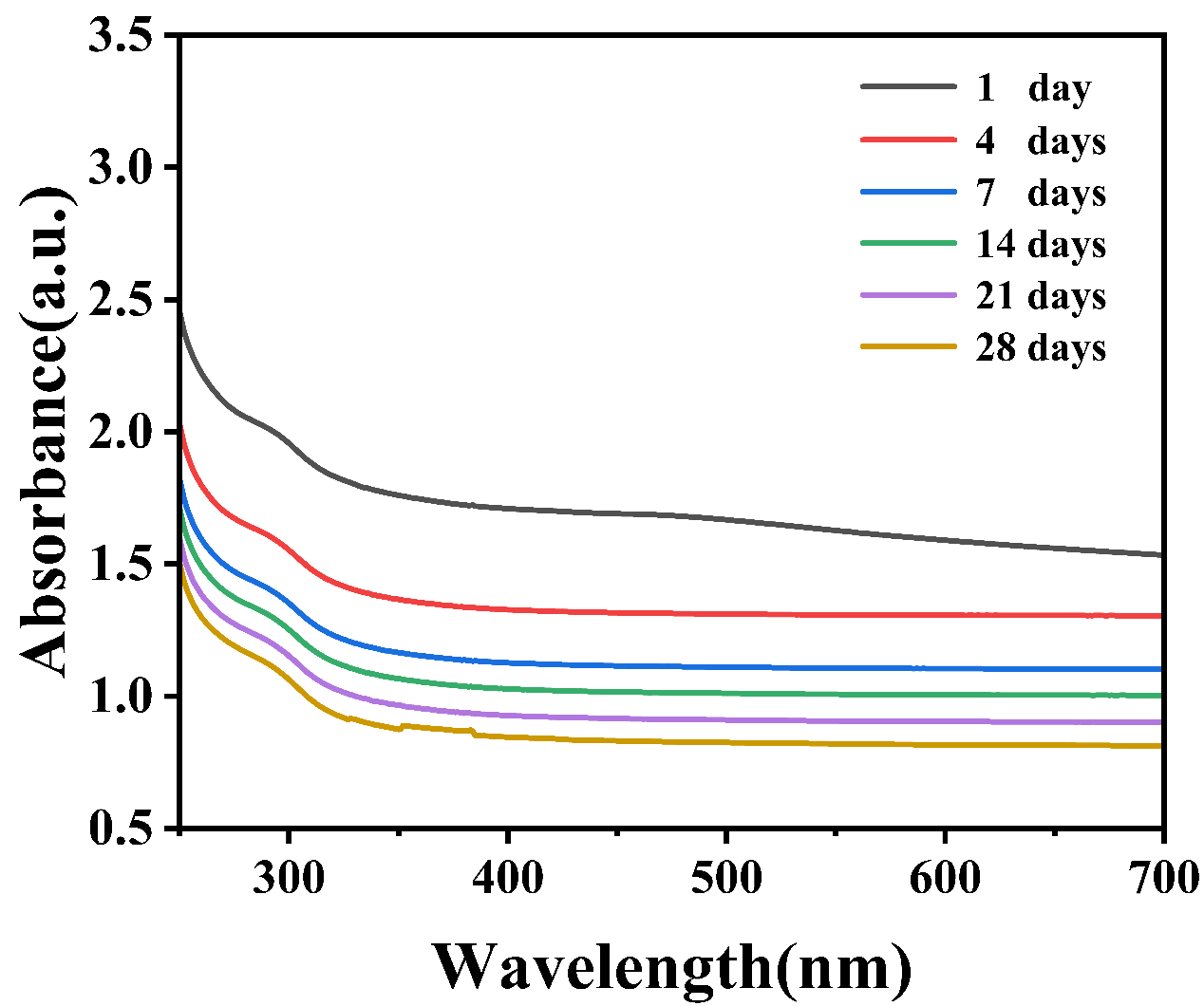


**Figure S3.** Characteristic UV-VIS spectral absorption peaks of BPNSs degraded over time on day 28.


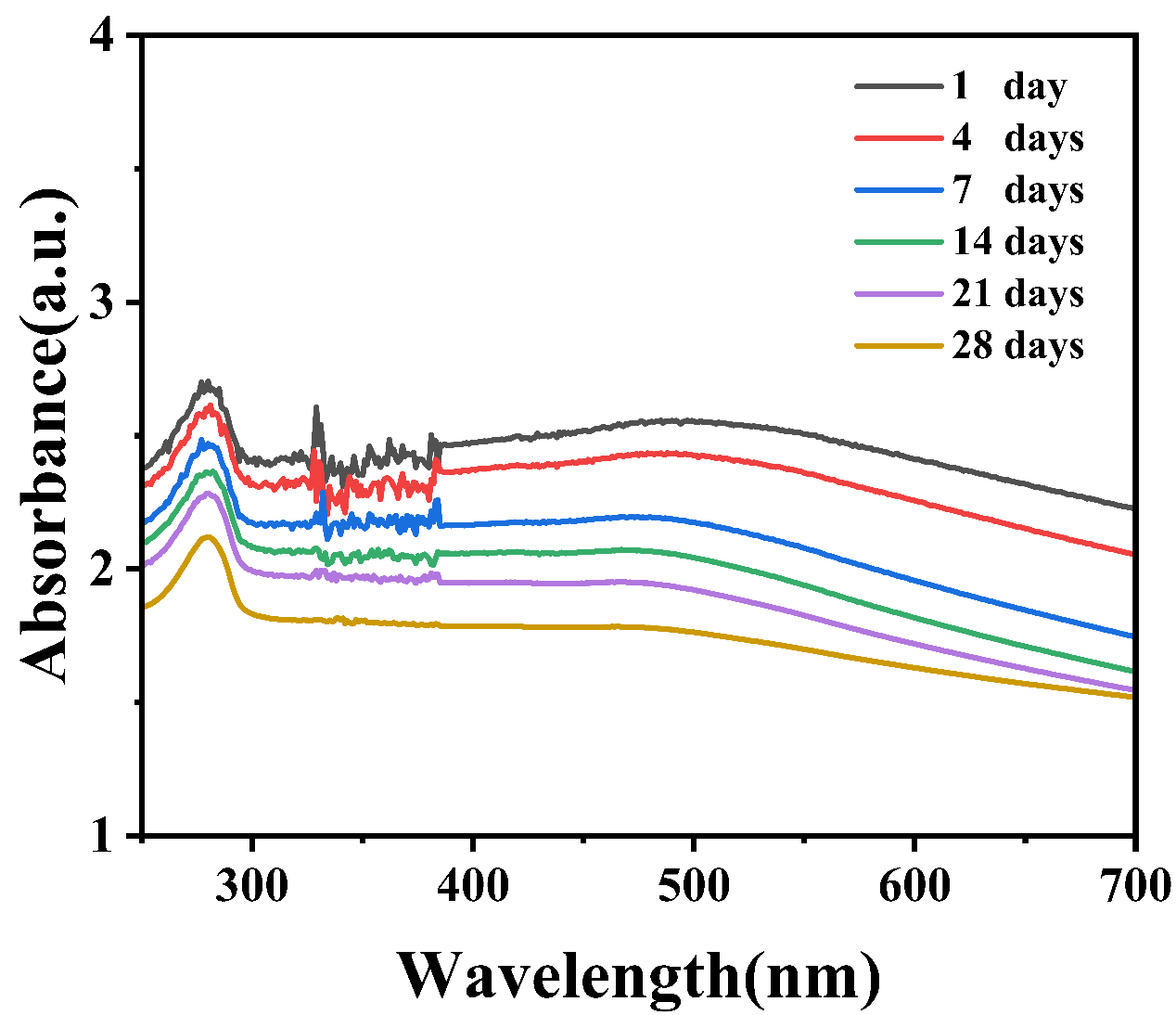


**Figure S4.** Characteristic UV-VIS spectral absorption peaks of pBP@Mg degraded over time on day 28.


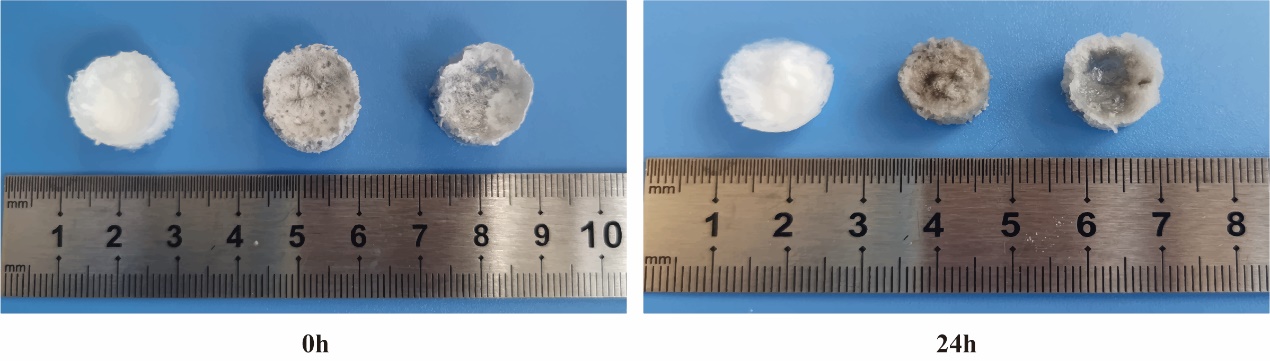


**Figure S5.** Digital photographs of 0h and 24h swelling of Gel, Gel-BP, and Gel-pBP@Mg.

| Samples | Conductivity (S/cm) |
| --- | --- |
| Gel-pBP@Mg-1 | 5.365×10^-4^ |
| Gel-pBP@Mg-2 | 5.031×10^-4^ |
| Gel-pBP@Mg-3 | 4.879×10^-4^ |
| Native myocardium tissue | 5×10^-5^-1.6×10^-3^ |

**Figure S6.** Gel-pBP@Mg four-point probe method resistivity detection.


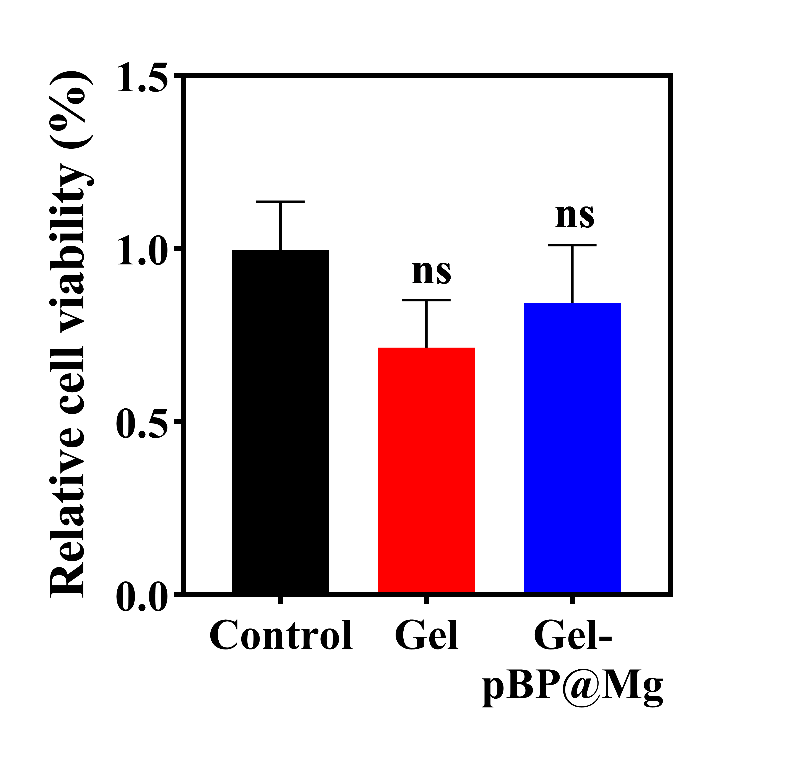


**Figure S7.** CCK-8 cell viability assay of Gel and Gel-pBP@Mg (n=3).


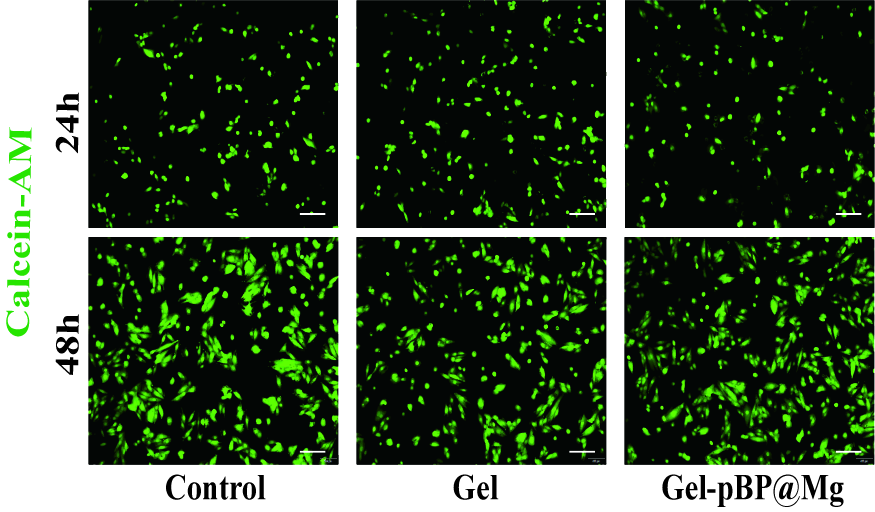


**Figure S8.** Calcein-AM staining after CMs culture on Gel and Gel-pBP@Mg for 24h and 48h.


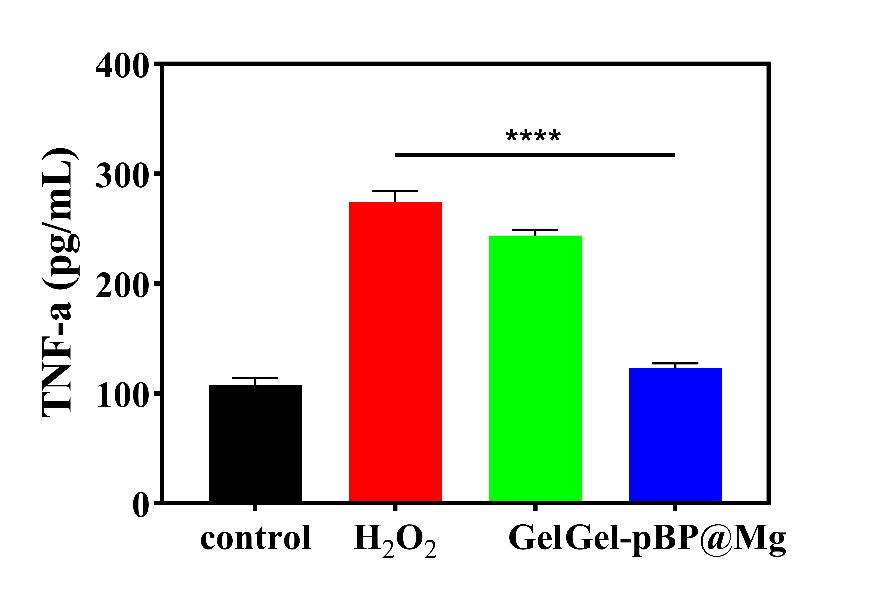


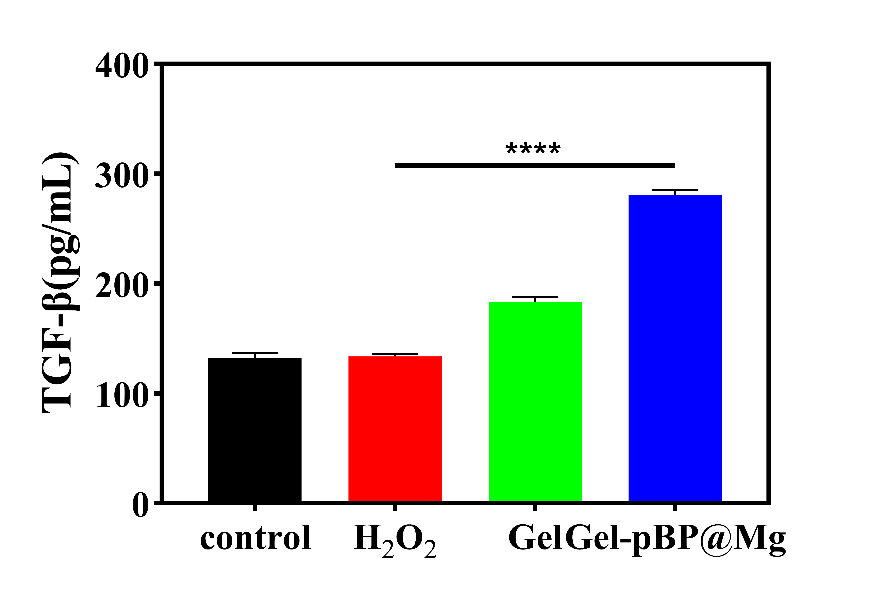


**Figure S9.** ELISA results of TNF-α and TGF-β in the supernatant of RAW264.7 cell culture medium in different treatment groups (n=7).


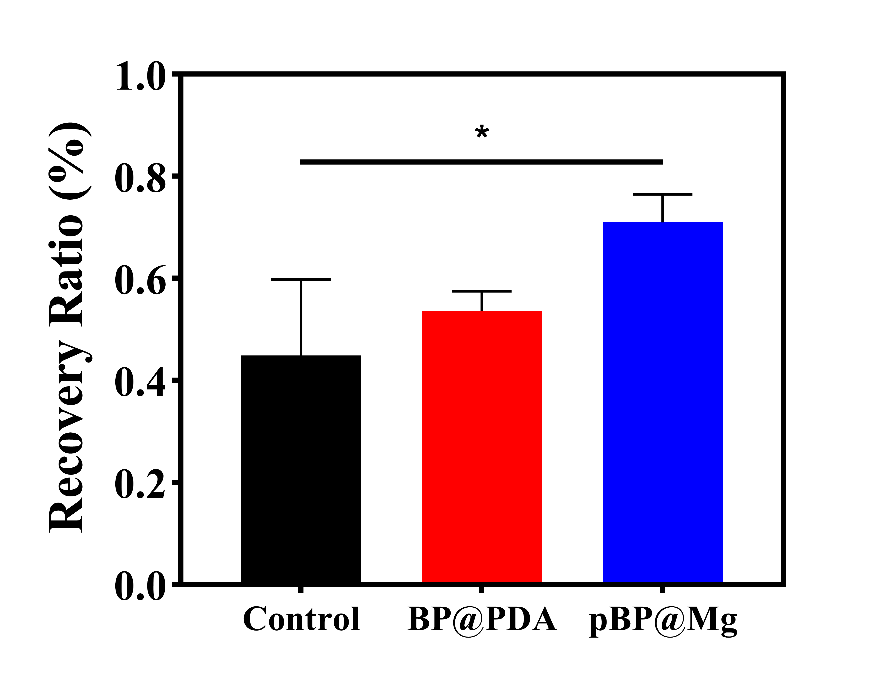


**Figure S10.** Quantitative statistical analysis of Control, BP@PDA, and pBP@Mg incubation for 24 h to promote HUVEC migration (n=3).


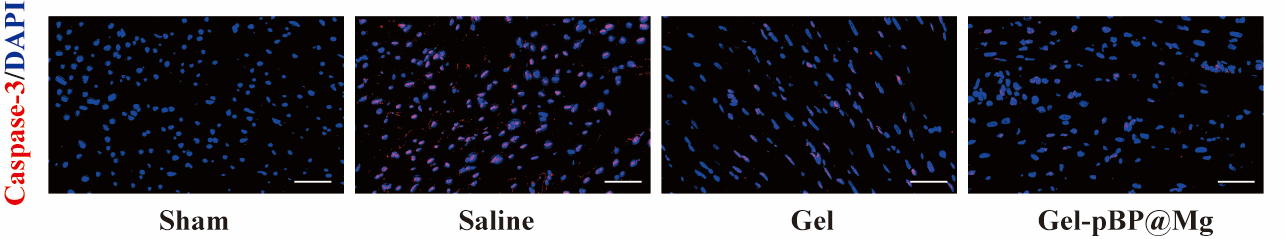


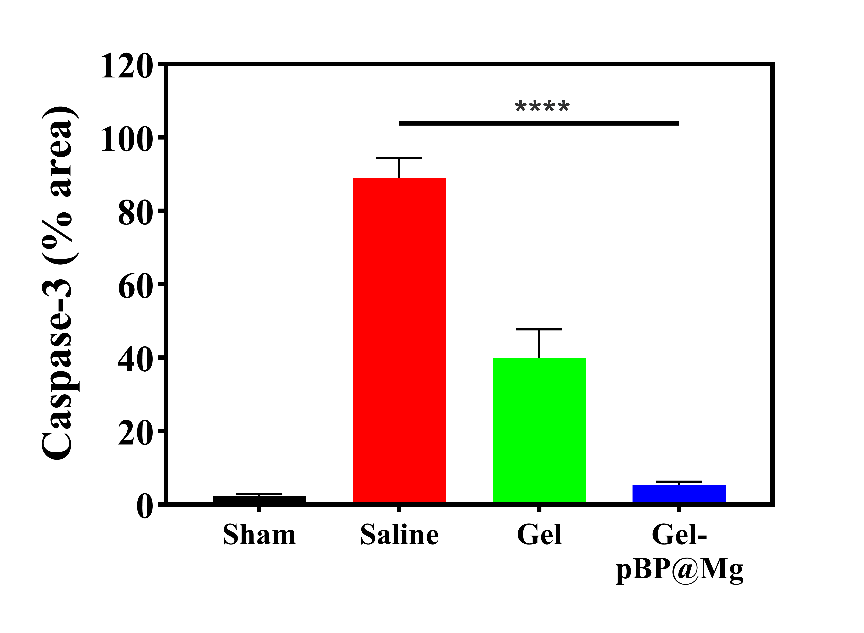


**Figure S11.** Caspase-3 immunofluorescence staining and quantitative statistical analysis on day 28 cardiac sections fabricated in Sham, Saline, Gel and Gel-pBP@Mg groups (n=4).


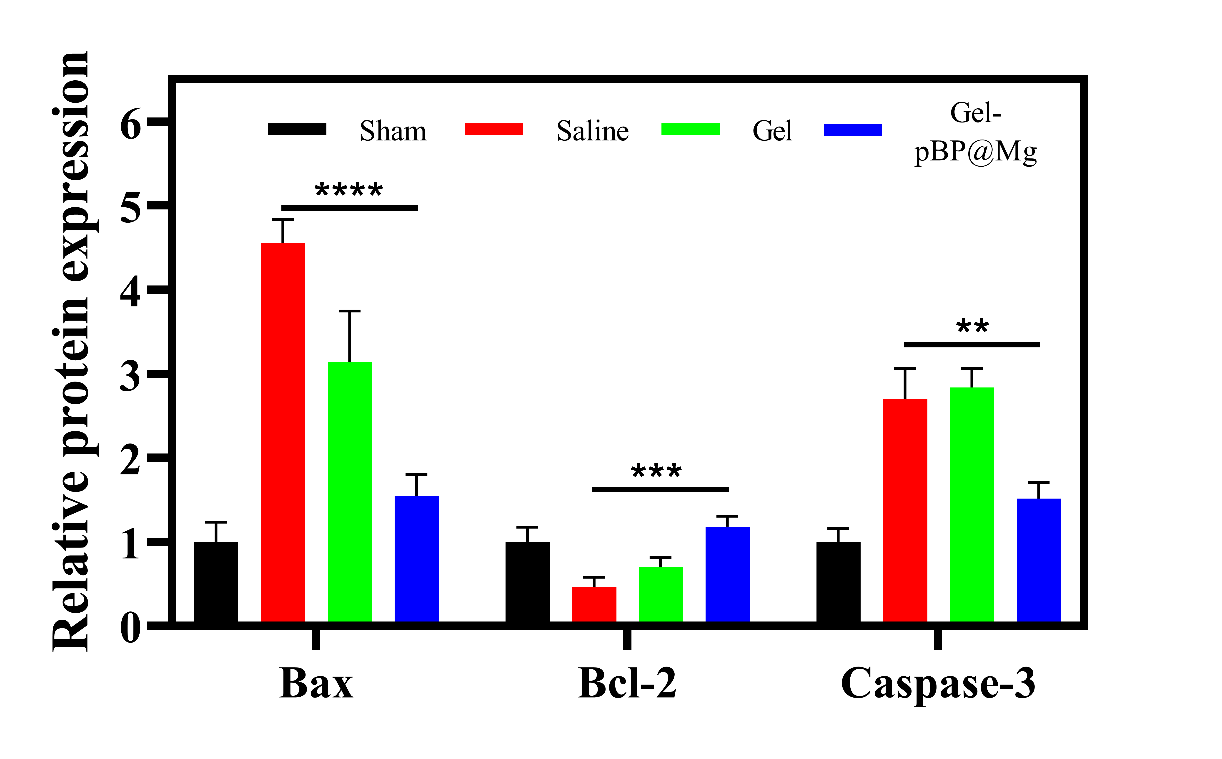


**Figure S12.** Statistical analysis of apoptosis pathway related protein expression (n=3).


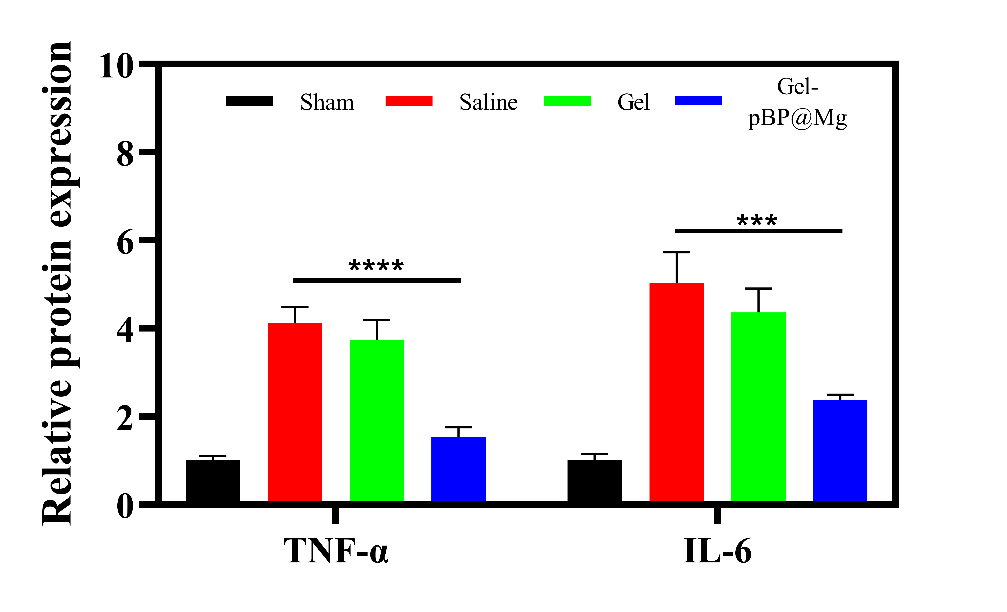


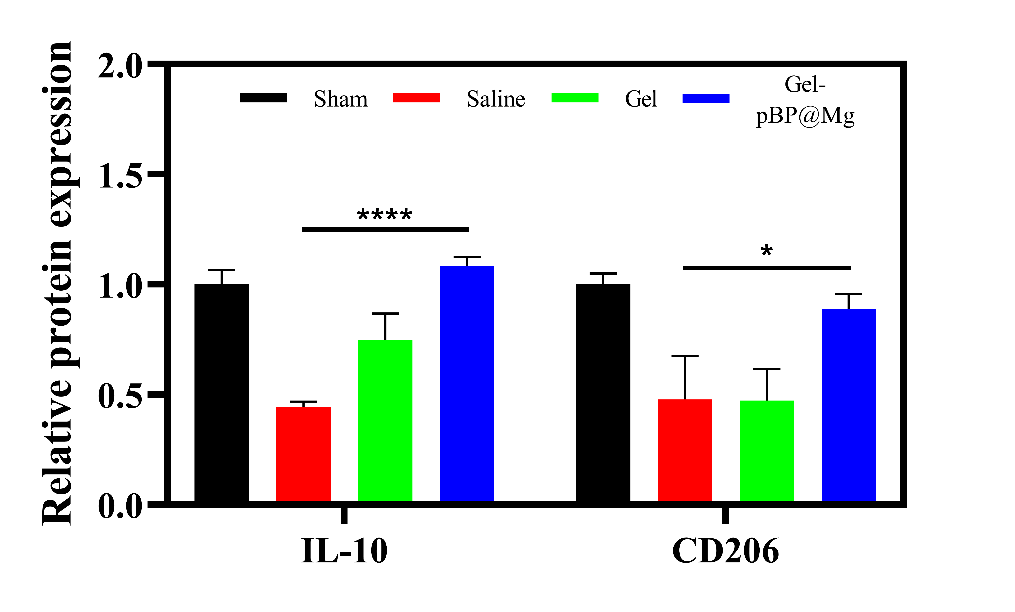


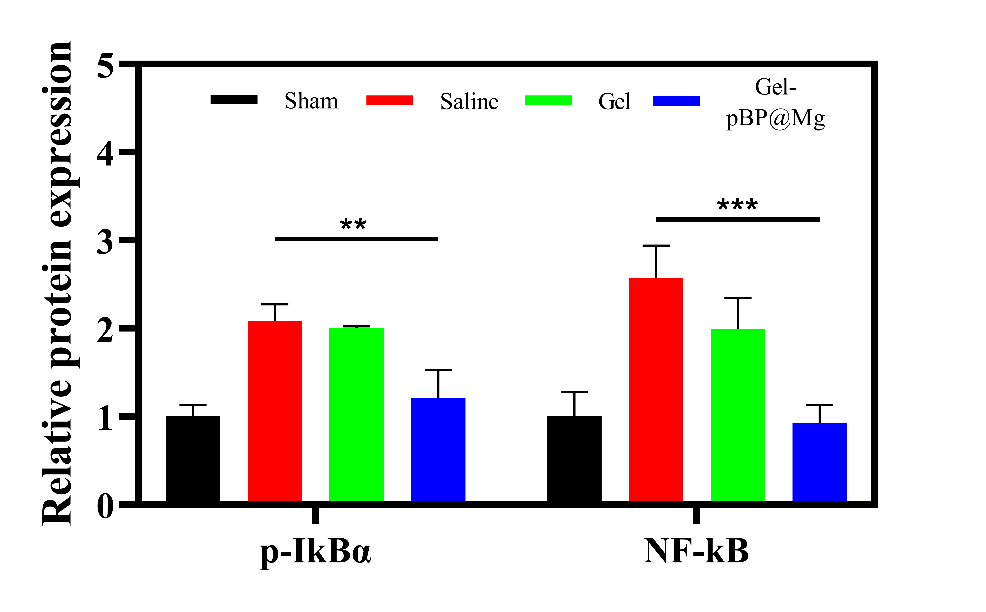


**Figure S13.** Statistical analysis of expression of inflammatory factor-related proteins in M1 and M2 phenotypic macrophages (n=3).


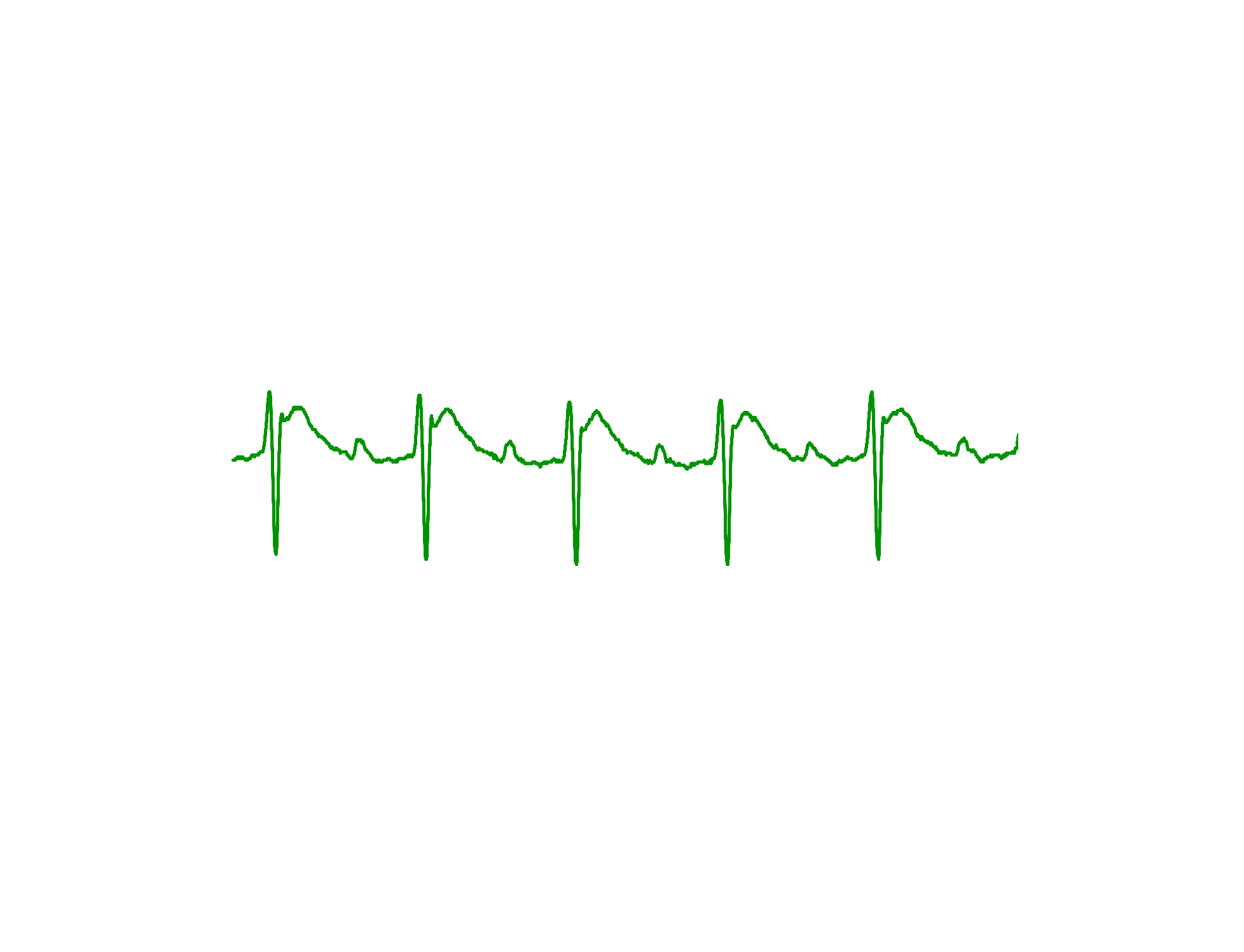


**Figure S14.** ECG detection of MI rats.


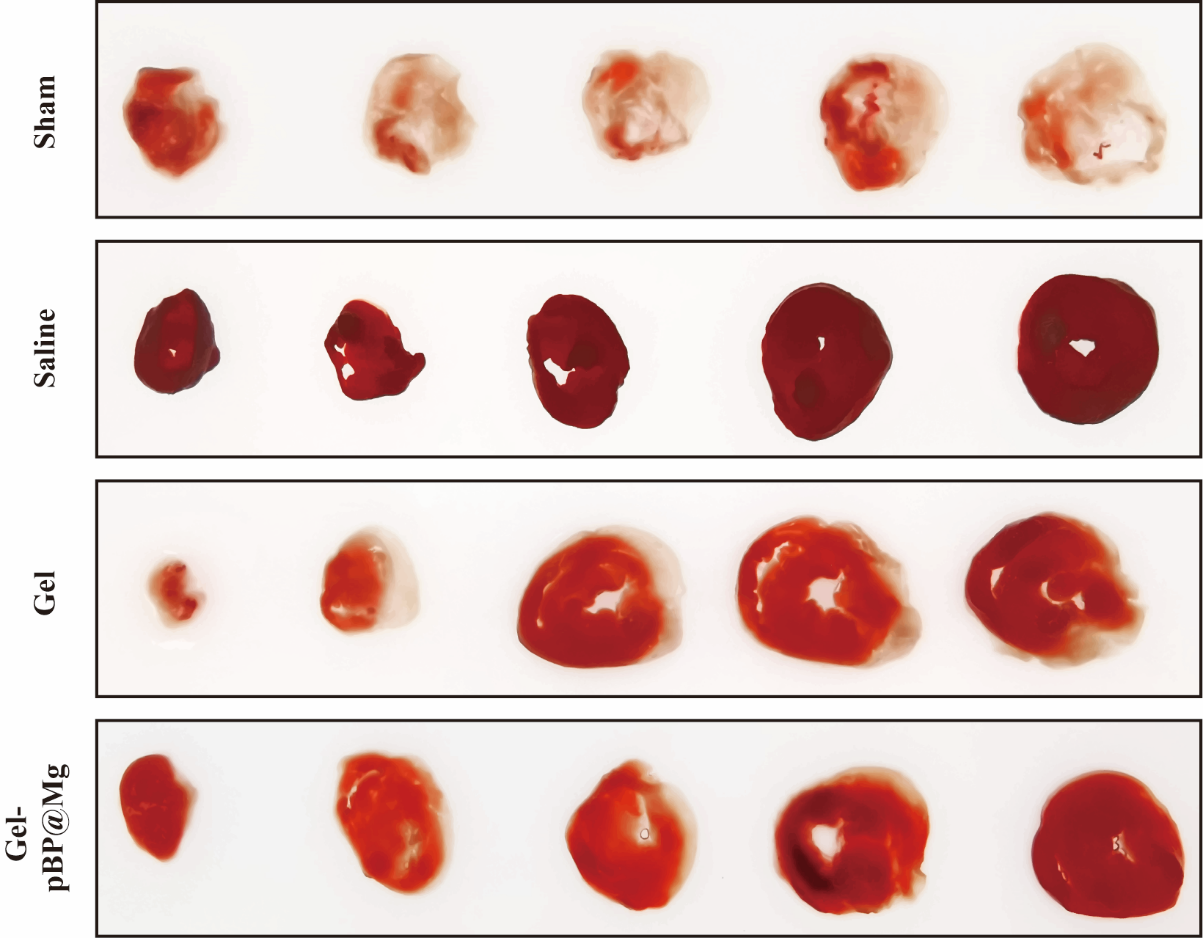


**Figure S15.** TTC staining digital images on day 28 cardiac tissue sections fabricated with Sham, Saline, Gel and Gel-pBP@Mg.
